# The 3′-Phosphoadenosine 5′-Phosphosulfate Transporters, PAPST1 and 2, Contribute to the Maintenance and Differentiation of Mouse Embryonic Stem Cells

**DOI:** 10.1371/journal.pone.0008262

**Published:** 2009-12-11

**Authors:** Norihiko Sasaki, Takuya Hirano, Tomomi Ichimiya, Masahiro Wakao, Kazumi Hirano, Akiko Kinoshita-Toyoda, Hidenao Toyoda, Yasuo Suda, Shoko Nishihara

**Affiliations:** 1 Laboratory of Cell Biology, Department of Bioinformatics, Faculty of Engineering, Soka University, Hachioji, Tokyo, Japan; 2 Department of Nanostructure and Advanced Materials, Graduate School of Science and Engineering, Kagoshima University, Kohrimoto, Kagoshima, Japan; 3 Laboratory of Bio-analytical Chemistry, College of Pharmaceutical Sciences, Ritsumeikan University, Kusatsu, Shiga, Japan; 4 Core Research for Evolutional Science and Technology (CREST) of Japan Science and Technology Agency (JST), Kawaguchi, Saitama, Japan; Oklahoma Medical Research Foundation, United States of America

## Abstract

Recently, we have identified two 3′-phosphoadenosine 5′-phosphosulfate (PAPS) transporters (PAPST1 and PAPST2), which contribute to PAPS transport into the Golgi, in both human and *Drosophila*. Mutation and RNA interference (RNAi) of the *Drosophila PAPST* have shown the importance of PAPST-dependent sulfation of carbohydrates and proteins during development. However, the functional roles of PAPST in mammals are largely unknown. Here, we investigated whether PAPST-dependent sulfation is involved in regulating signaling pathways required for the maintenance of mouse embryonic stem cells (mESCs), differentiation into the three germ layers, and neurogenesis. By using a yeast expression system, mouse PAPST1 and PAPST2 proteins were shown to have PAPS transport activity with an apparent *K_m_* value of 1.54 µM or 1.49 µM, respectively. RNAi-mediated knockdown of each *PAPST* induced the reduction of chondroitin sulfate (CS) chain sulfation as well as heparan sulfate (HS) chain sulfation, and inhibited mESC self-renewal due to defects in several signaling pathways. However, we suggest that these effects were due to reduced HS, not CS, chain sulfation, because knockdown of mouse *N-deacetylase/N-sulfotransferase*, which catalyzes the first step of HS sulfation, in mESCs gave similar results to those observed in *PAPST*-knockdown mESCs, but depletion of CS chains did not. On the other hand, during embryoid body formation, *PAPST*-knockdown mESCs exhibited abnormal differentiation, in particular neurogenesis was promoted, presumably due to the observed defects in BMP, FGF and Wnt signaling. The latter were reduced as a result of the reduction in both HS and CS chain sulfation. We propose that PAPST-dependent sulfation of HS or CS chains, which is regulated developmentally, regulates the extrinsic signaling required for the maintenance and normal differentiation of mESCs.

## Introduction

Embryonic stem cells (ESCs) [1], [2] are promising tools for biotechnology and possess key features that should allow their exploitation in the development of cell replacement therapies [3]. To exploit the potential of ESCs for therapeutic purposes, a better understanding of the molecular mechanisms that control the pluripotency and differentiation of ESCs is required. The factors that control the pluripotency of mouse ESCs (mESCs) are increasingly being defined and the regulation of pluripotency requires a combination of extrinsic and intrinsic factors [4], [5]. A number of the intrinsic factors, such as Oct3/4 and Nanog, have been identified [6]. Recent studies have shown that mESCs and human ESCs (hESCs) maintain their pluripotency using different extrinsic factors.

Leukemia inhibitory factor (LIF) [7], [8], which is one of the known extrinsic factors, plays an important role in maintaining the self-renewal of mESCs via the activation of STAT3 [9]–[12] and induction of c-Myc [13]. One of the other extrinsic factors involved in the maintenance of mESC self-renewal is bone morphogenic protein 4 (BMP4). BMP4 acts in synergy with LIF to maintain self-renewal via the Smad-mediated induction of *Id* (*inhibitor of differentiation*) gene expression [14] and inhibition of p38 mitogen-activated protein kinase [15]. Wnt/β-catenin signaling also plays a role in the regulation of self-renewal of both mESCs and hESCs and this signaling is independent of LIF/STAT3 signaling [16]–[19]. It has been demonstrated that signaling by the canonical Wnt pathway increases and maintains Nanog expression [16]–[18]. Thus, the activation of Nanog by Wnt/β-catenin signaling can sustain ESC self-renewal without the use of feeder cells or treatment with LIF [16], [17].

To enable the production of differentiated cells of a specific lineage, the mechanism of regulation of extrinsic signaling in ESCs has been investigated by applying knowledge obtained from analysis of the early mouse embryo. It is known that several extrinsic factors, such as BMP, fibroblast growth factor (FGF) and Wnt, play important roles in the differentiation of mESCs, in addition to their involvement in self-renewal [20]. BMP/Smad signaling is essential for the decision between ectodermal and mesodermal fates. It has been demonstrated that antagonism of BMP/Smad signaling, for example by exposure of mESCs to Noggin or by transfection with a Noggin-encoding plasmid, promotes neurectodermal differentiation via embryoid body (EB) formation [21], [22]. FGF4 is produced in an autocrine fashion in mESCs and FGF4/extracellular signal-regulated kinase (ERK) signaling contributes to differentiation into neural and mesodermal lineages [23]. Wnt/β-catenin signaling inhibits neural differentiation via EB formation: either inactivation of the adenomatous polyposis coli (APC) protein, which regulates the activity of β-catenin, or the introduction of a dominant active form of β-catenin results in the inhibition of neural differentiation in mESCs [24]. Furthermore, the Wnt antagonist *Sfrp2* is expressed during the neural differentiation of EBs and expression of *Sfrp2* enhances neuronal differentiation [25].

Sulfation is an essential modification of many carbohydrates and proteins, and is necessary for normal growth and development. In higher organisms, all sulfation reactions require the high energy sulfate donor 3′-phosphoadenosine 5′-phosphosulfate (PAPS) [26]. PAPS is synthesized in the cytosol and nucleus by PAPS synthetase [27], [28] and is subsequently translocated into the Golgi via the PAPS transporter (PAPST) [29]–[32] to serve as a substrate for sulfotransferases. Recently, we identified and characterized two homologues of PAPST (PAPST1 and PAPST2) in both human and *Drosophila* [29], [31], [32].

Mutations in the *Drosophila PAPST1* gene, *slalom*, are associated with defects in multiple signaling pathways, including Wnt/Wingless (Wg) and Hedgehog (Hh) signaling, and in the determination of the embryonic dorsal/ventral axis [30]. These defects are suggested to be due to a lack of sulfation of heparan sulfate (HS) chains. HS, a sulfated glycosaminoglycan (GAG), is present ubiquitously as a cell surface proteoglycan. HS chains are known to play crucial roles in the regulation of several signaling pathways by controlling the binding of various extracellular signaling molecules, such as members of the FGF family, Wnt/Wg, Hh and BMP, to their cognate receptors [33]. Recently, we have also suggested that the second *Drosophila* PAPST, dPAPST2, contributes to signaling by Hh and Decapentaplegic by controlling HS chain sulfation [32]. It has been reported that mutants of the zebrafish *PAPST1* gene, *pinscher*, have cartilage defects that are analogous to those found in the zebrafish *dackel* mutant, which is HS chain defective [34]. The above genetic experiments have established that sulfation of GAGs is essential for normal development and that the regulation of sulfation is extremely important.

In mammals, the importance of HS chains during development has been demonstrated by the analysis of mutations in enzymes required for HS chain modification [33], [35]–[38]. Recently, we have demonstrated that HS chains contribute to the self-renewal and pluripotency of mESCs and that this role involves the regulation of Wnt/β-catenin signaling [17]. Other groups have reported that HS chains contribute to the differentiation of mESCs into mesodermal and neurectodermal lineages [39], [40]. Thus, there is evidence that HS chains have essential functions in development including in ESCs. However, the significance during development of the PAPST-dependent sulfation of either HS or other sulfated carbohydrates, such as chondroitin sulfate (CS), which is another major sulfated GAG and is implicated in the signaling pathway of heparin-binding growth factors [41], [42], is not understood well.

In the present study, we analyzed the function of *PAPST1* and *PAPST2* by performing RNA interference (RNAi). Although the knockdown (KD) efficiency was less than 100%, we used this method rather than performing gene knockouts because, in addition to the direct effects of gene knockouts, secondary effects may also be observed that are caused by adaptation of the cells during long-term culture. For example, the expression of a novel gene might be induced that has secondary effects on the mESCs. If, as in the case of the *PAPST* genes, the RNAi targets are essential for cell survival and proliferation, analysis of the knockout cells may be complicated by cell death. In fact, knockout of some genes that are related to HS sulfation, e.g., *6-O-endosulfatase*, *C5-epimerase* and *HS2ST*, leads to a number of unexpected changes in the structure of sulfated GAGs, presumably due to secondary effects [36], [38], [43].

Our current understanding is that sulfated carbohydrates contribute to the maintenance and differentiation of ESCs by regulating the binding of extrinsic factors and subsequent signal transduction. In this study, we investigated the contribution of PAPST-dependent sulfation to the regulation of mESC self-renewal and pluripotency, differentiation into the three germ layers, and neurogenesis. First, we confirmed that the mouse solute carrier family 35B2 (SLC35B2) and SLC35B3 proteins, namely mouse PAPST1 and PAPST2, both exhibited PAPS transport activity. Then we showed that knockdown of either *PAPST1* or *PAPST2* in mESCs reduced the self-renewal and proliferation of the cells even in the presence of LIF and serum. These effects are likely to be due to the reduction of HS chain sulfation, because knockdown of mouse *N-deacetylase/N-sulfotransferase* (*NDST*), which encodes the enzyme responsible for the first step of HS sulfation, in mESCs resulted in similar effects but depletion of CS chains did not. Both *PAPST1*- and *PAPST2*-KD mESCs exhibited abnormal differentiation during EB formation. In particular, neurogenesis was promoted due to the reduction of both HS and CS chain sulfation. We highlight here the importance of PAPST-dependent sulfation for the maintenance of the self-renewal and pluripotency of mESCs and also the normal differentiation of EBs.

## Results

### Both Mouse *PAPST1* and *PAPST2* Are PAPS Transporter Genes

The human *PAPST1* and *PAPST2* genes are members of SLC35B. The mouse proteins SLC35B2 and SLC35B3 (NCBI accession numbers NP_082938 and NP_598821, respectively) share 82.41% and 83.54% homology with the human orthologs PAPST1 and PAPST2, respectively. Hydrophobicity analyses of the amino acid sequences using the SOSUI system (Mitsui Knowledge Industry Co., Ltd.) revealed that mouse PAPST1 and PAPST2 were type III transmembrane proteins with eight and nine transmembrane domains, respectively. The mouse *PAPST1* and *PAPST2* genes consist of four and ten exons, respectively, and both were expressed ubiquitously in all organs (Data not shown).

**Figure 1:**
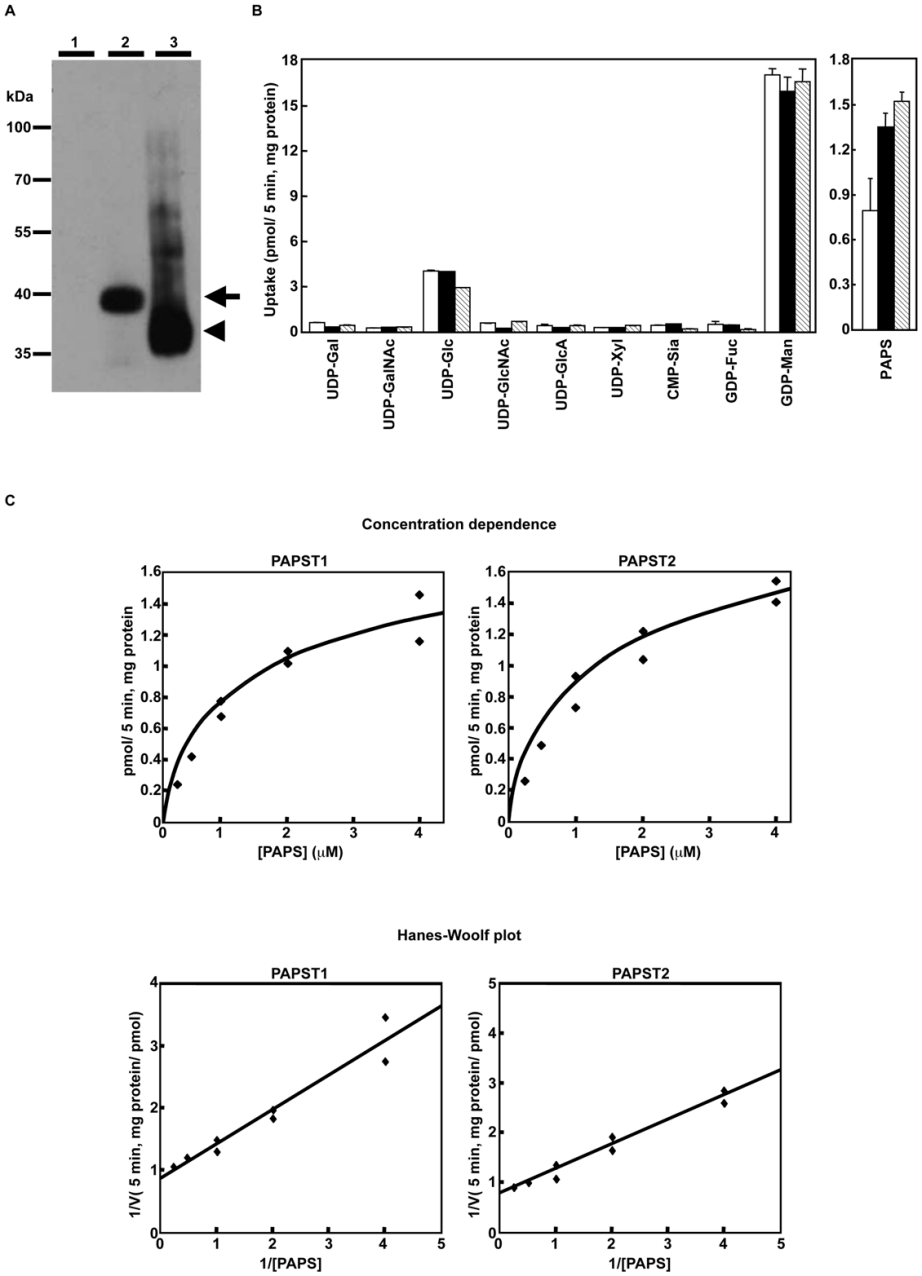
Both mouse *PAPST1* and *PAPST2* encode PAPS transporter proteins. (A) Expression state of PAPST1 and PAPST2 proteins in the Golgi-enriched fraction. Western blot analysis of the P100 fractions prepared from yeast cells expressing either the mock vector (*lane 1*), HA-tagged *PAPST1* (*lane 2*) or HA-tagged *PAPST2* (*lane 3*). An aliquot of 5 µg of protein from the control cells and cells expressing HA-tagged *PAPST1* or 0.5 µg of protein from the cells expressing HA-tagged *PAPST2* was loaded. The *arrow* and *arrowhead* indicate HA-tagged PAPST1 and HA-tagged PAPST2, respectively. (B) Substrate specificity of PAPST1 and PAPST2. Each P100 fraction was incubated in 50 µl of reaction buffer containing 1 µM labelled substrate at 32°C for 5 min, and the radioactivity incorporated was measured. The indicated values are the mean±SD obtained from two independent experiments (*open bars*, Mock; *solid bars*, *PAPST1*; *hatched bars*, *PAPST2*). (C) Substrate concentration dependence. Each P100 fraction was incubated in 50 µl of reaction buffer containing different concentrations of [^35^S]PAPS at 32°C for 5 min, and the radioactivity incorporated was measured. Specific incorporation was calculated by subtracting the value for the mock transfection from each of the values obtained. *Lower panel*, the Hanes-Woolf plot used to determine the *Km* value is shown.

The substrate specificity of the mouse PAPST1 and PAPST2 proteins was examined using a yeast expression system in a manner similar to that used to investigate human PAPST1 and PAPST2 [29], [31]. The coding sequence for hemagglutinin (HA)-tagged *PAPST1* or HA-tagged *PAPST2* was inserted into the yeast expression vector YEp352GAP-II. The constructs were then introduced into W303-1a yeast to allow preparation of the Golgi-enriched P100 membrane fraction that contained the mouse PAPST1 or PAPST2 protein. The HA-tagged PAPST1 and PAPST2 proteins were detected in the yeast P100 membrane fraction by Western blotting using an antibody against the HA epitope tag (Figure 1A). The substrate specificity of the PAPST1 and PAPST2 proteins was examined using the P100 membrane fraction and radiolabeled substrates. The P100 membrane fractions prepared from yeast cells that expressed PAPST1 or PAPST2 showed PAPS transport activity that was significantly higher than that observed in the control cells (Figure 1B). The dependence of PAPS transport by PAPST1 and PAPST2 on substrate concentration is shown in Figure 1C. Both PAPST1 and PAPST2 showed a saturable PAPS transport activity with apparent *K_m_* values that were estimated to be 1.54 µM and 1.49 µM, respectively.

### Sulfation of Several Substrates Is Reduced by Knockdown of *PAPST* mRNA

**Figure 2:**
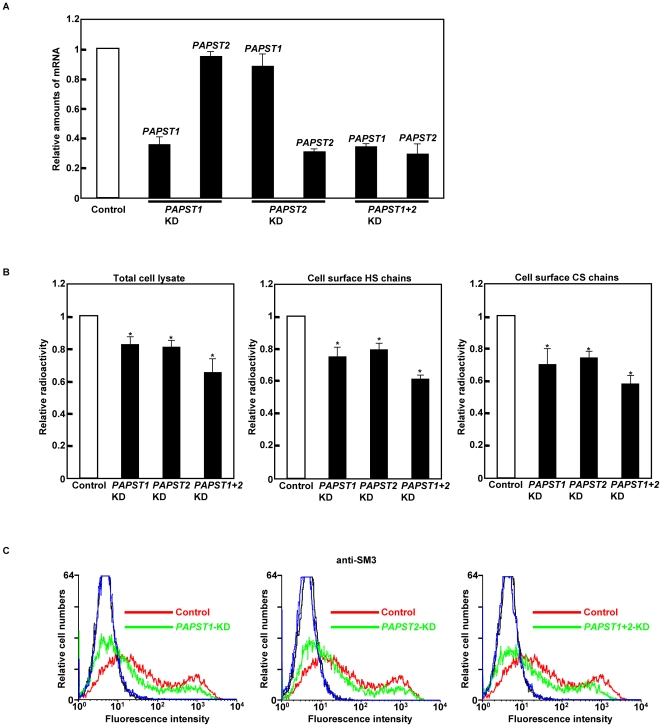
Knockdown of *PAPST1* or *PAPST2* mRNA induced reduction of sulfation in mESCs. (A) Real time PCR analysis of cells 2 days after transfection. Relative amounts of *PAPST* mRNA were calculated after normalization to *β-actin* mRNA in the same cDNA. The results are shown after normalization against the values obtained with control cells (value = 1). The values shown are the means±SD of three independent experiments. (B) Metabolic labeling analysis. The results of total sulfate incorporation into cellular proteins and sulfate incorporation into cell surface HS and CS chains are shown after normalization against the values obtained with control cells (value = 1). The values shown are the means±SD of three independent experiments and significant values are indicated; *P<0.01, in comparison to the control. (C) FACS analysis of cells 3 days after transfection using an anti-SM3 antibody (black and blue lines represent the IgM isotype control for control and *PAPST*-KD cells, respectively). Three independent experiments were performed and representative results are shown.

To examine the effects of reduced sulfation in mESCs, we knocked down the expression of either *PAPST1* or *PAPST2* mRNA by RNAi. Real time PCR performed 2 days after transfection of the small interfering RNAs (siRNAs) showed that the level of *PAPST1* or *PAPST2* mRNA was reduced to approximately 40% of that in control cells (Figure 2A). The level of *PAPST1* mRNA was unaffected by the *PAPST2* siRNAs, and similarly the level of *PAPST2* mRNA was not decreased by the *PAPST1* siRNAs (Figure 2A), which confirmed the specificity of the targeting sequences. When the cells were transfected with both *PAPST1* and *PAPST2* siRNAs (*PAPST1*+*2*-KD cells), the level of both *PAPST1* and *PAPST2* mRNA was reduced to approximately 40% of that in control cells (Figure 2A). The results shown in this paper were obtained using the *PAPST1-1* and *PAPST2-1* siRNAs, however we obtained similar results using other siRNA sequences against *PAPST1* and *PAPST2* (Data not shown). For all experiments, we examined the effects of the knockdowns in the R1 mESC line first using two types of siRNA expression plasmid and then confirmed the effects in the E14TG2a line using a single siRNA expression plasmid.

We first examined endogenous PAPS transport activity in the mESCs. PAPS transport activities in both *PAPST1*- and *PAPST2*-KD cells were reduced to approximately 80% of that of control cells, which confirms that both the *PAPST1* and *PAPST2* genes encode a PAPST protein (Figure S1). We next determined by metabolic labeling whether total sulfate incorporation into cellular proteins in mESCs was reduced by knockdown of *PAPST1* or *PAPST2* mRNA. The radioactivity incorporated into cellular proteins in both *PAPST1*- and *PAPST2*-KD cells was approximately 80% of that incorporated in control cells (Figure 2B). Furthermore, sulfation of cell surface GAGs, such as HS and CS chains, in both *PAPST1*- and *PAPST2*-KD cells was significantly reduced to approximately 60–70% of that of control cells (Figure 2B). The *PAPST1*+*2*-KD cells showed the lowest incorporation value, indicating additive effect from reduction of both PAPST1 and PAPST2. In *PAPST1*-transfected MDCK II cells, chain length of GAGs was changed compared with non-transfected cells [44]. So, we examined length of HS and CS chains in both *PAPST1*- and *PAPST2*-KD cells, but no detectable differences between *PAPST*-KD cells and control cells were detected (Data not shown).

We performed FACS analysis to examine other sulfated substrates. Sulfatide SM3 (SO_3_-3Galβ1-4Glcβ1-1Cer), one of the sulfated glycolipids, was reduced slightly in both *PAPST1*- and *PAPST2*-KD cells compared to that observed in control cells (Figure 2C). Other sulfated glycans and glycolipids, such as 3′-sulfo-Le^a^ (SO_3_-3Galβ1-3(Fucα1-4)GlcNAc), human natural killer-1 (HNK-1) carbohydrate (SO_3_-3GlcAβ1-3Galβ1-4GlcNAc) and sulfatide SM4 (SO_3_-3Galβ1-1Cer), were not detected by FACS analysis (Data not shown), showing that such molecules were not present on the surface of mESCs.

These results demonstrate that both PAPSTs contribute comparably to the sulfation of proteins and several carbohydrates.

### PAPST1- and PAPST2-Dependent Sulfation of HS Chains but Not CS Chains Is Important for the Self-Renewal and Proliferation of mESCs

**Figure 3:**
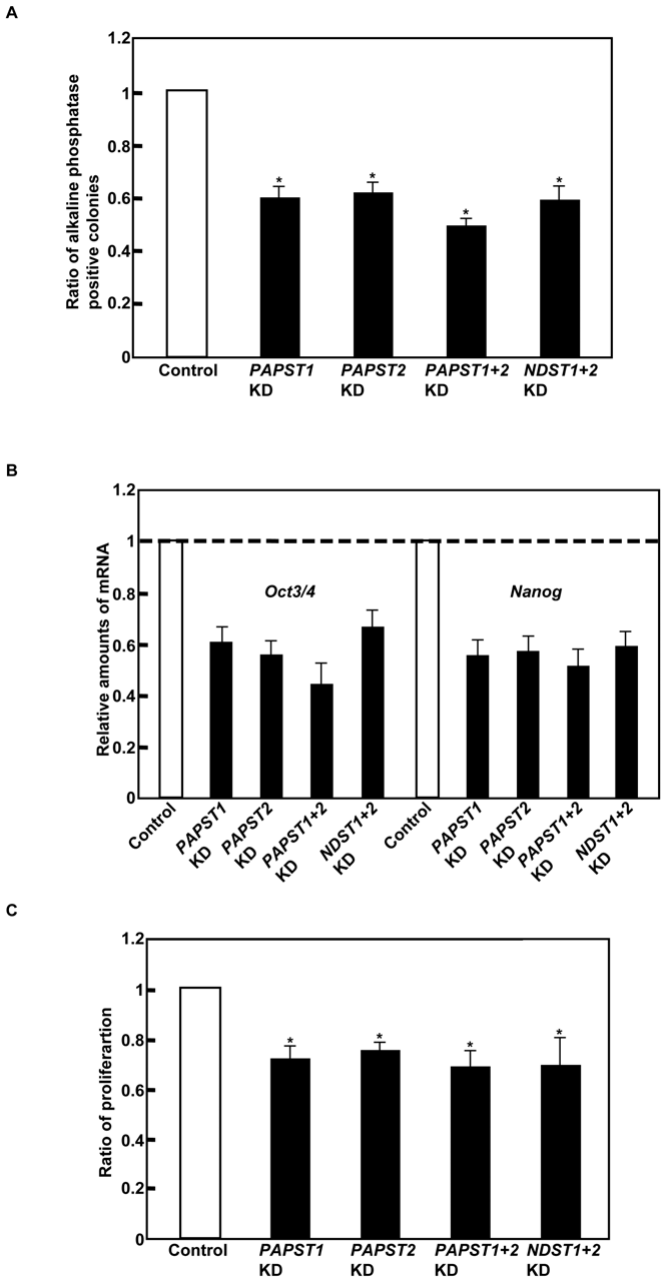
Both *PAPST1*- and *PAPST2*-KD cells showed decreased potential for self-renewal and proliferation. (A) Self-renewal assay. The ratio of alkaline phosphatase positive colonies is shown after normalization against the ratio obtained with control cells (value = 1). Approximately 70% of the colonies derived from the control cells remained in an undifferentiated state in feeder-free culture. The values shown are the means±SD from three independent experiments and significant values are indicated; *P<0.01, in comparison to the control. (B) Real time PCR analysis of undifferentiated state markers in the cells 4 days after transfection. The results are shown after normalization against the values obtained for control cells (value = 1). The values shown are the means±SD from two independent experiments. (C) Proliferation assay. The ratio of proliferation 48 h after culture is shown after normalization against the values obtained with control cells (value = 1). The values shown are the means±SD from three independent experiments and significant values are indicated; *P<0.01, in comparison to the control.

We performed colony assays with *PAPST1*- and *PAPST2*-KD cells to determine whether reduced sulfation affected self-renewal. The number of colonies derived from either *PAPST1*- or *PAPST2*-KD cells that remained in an undifferentiated state fell to approximately 60% of the number from control cells even in the presence of LIF and serum in clonal density culture, showing reduction of self-renewal in *PAPST*-KD cells (Figure 3A). Furthermore, the most of the *PAPST1*- and *PAPST2*-KD cells exhibited a flattened, differentiated morphology in normal density culture four days after transfection (Figure S2A). These results are supported by the reduced expression of *Oct3/4* and *Nanog*, markers of the undifferentiated state, and up-regulated expression of extraembryonic endoderm (ExE) lineage markers, *Gata6* (primitive endoderm), *LamininB1* (parietal endoderm) and *Bmp2* (visceral endoderm) (Figure 3B and S2B). The above results demonstrate that both PAPST1- and PAPST2-dependent sulfation is important for the maintenance of the undifferentiated state and pluripotency of mESCs.

Next we examined proliferation. As shown in Figure 3C, the proliferation of both *PAPST1*- and *PAPST2*-KD cells decreased significantly compared to that of control cells. We have reported previously that HS chains contribute to the self-renewal and proliferation of mESCs [17], but the contribution of CS chains is unknown. Depletion of CS chains by treatment with chondroitinase ABC (ChABC) did not affect self-renewal and proliferation (Figure S3A and B), which demonstrated that CS chains do not contribute to these processes in mESCs. Furthermore, we examined whether a specific reduction in HS chain sulfation would result in similar defects to those observed in *PAPST*-KD cells. In mESCs, only *NDST1* and *NDST2* are expressed equally [45]. Therefore, we knocked down both *NDST1* and *NDST2* to avoid functional compensation. The effects of the knockdown were confirmed by RT-PCR analysis and metabolic labeling. In the *NDST1*+*2*-KD cells, the levels of both *NDST1* and *NDST2* mRNA were reduced to 39.5±4.9% and 34.0±2.8% of those in control cells, respectively, and sulfation of HS chains was reduced to 69.6±7.3% of that in control cells. As shown in Figure 3 and S2B, *NDST1+2*-KD cells exhibited similar defects in self-renewal and proliferation as the *PAPST*-KD cells. Thus, we conclude that the defects observed in *PAPST*-KD cells are due to a reduction in HS chain sulfation. In addition, the knockdown of both *PAPST1* and *PAPST2* together had an additive effect on self-renewal, and it is likely that the further reduction of HS sulfation observed in the *PAPST1*+*2*-KD cells, as shown in Figure 2B, was responsible for this additive effect.

Taken together, these results demonstrate that the reduction of PAPST1- and PAPST2-dependent sulfation inhibit both self-renewal and proliferation of mESCs, and this is presumably due to reduced levels of HS chain-dependent signaling.

### The Reduction of PAPST-Dependent Sulfation Down-Regulates Several Signaling Pathways in mESCs

**Figure 4:**
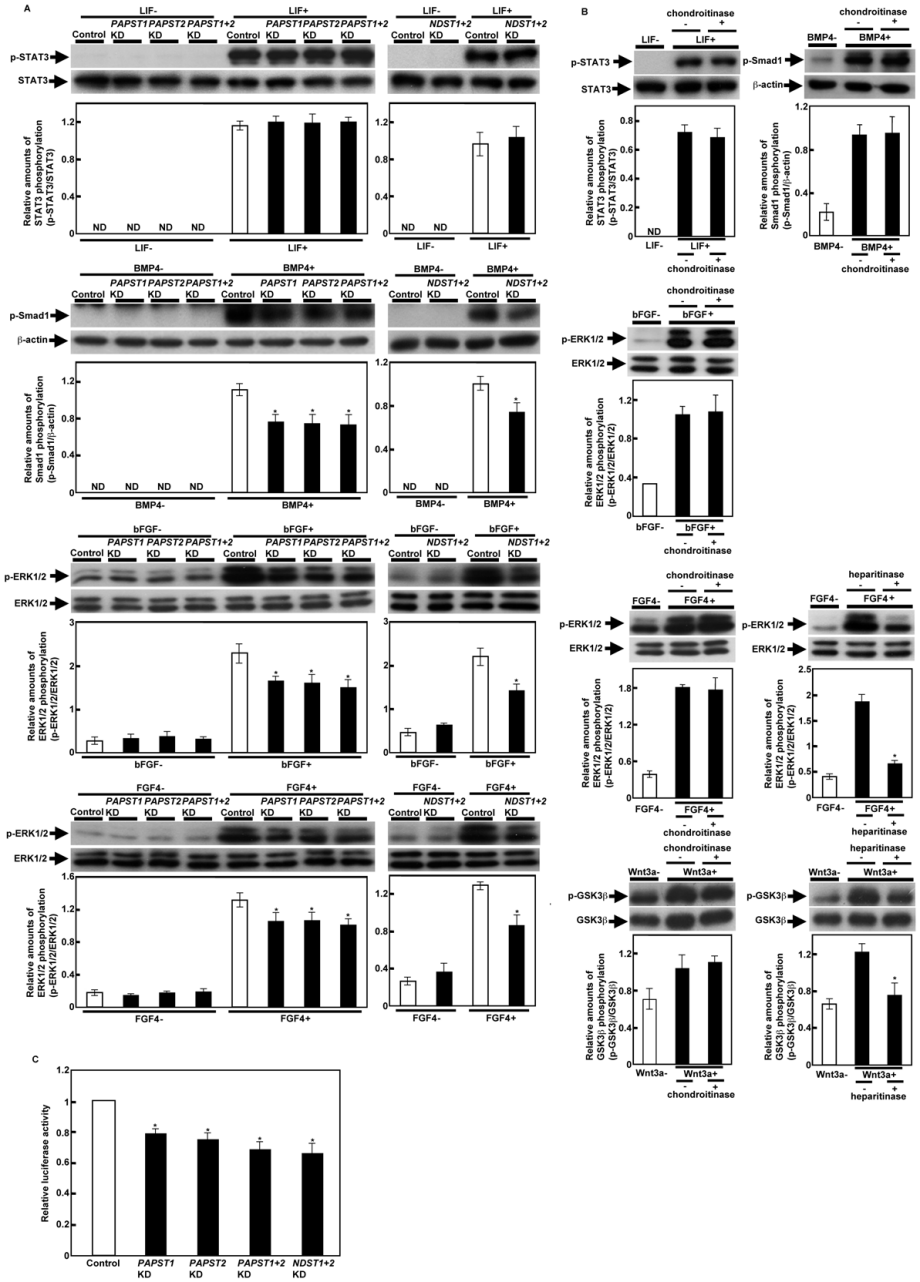
Signaling by specific factors was decreased in *PAPST*-KD cells, but not in CS chain-depleted cells. (A) and (B) Western blot analysis of cells stimulated with the extrinsic factors. Cell lysate was prepared as described in Materials and Methods. Two independent experiments were performed and representative results are shown. The histograms show mean densitometric readings±SD of the phosphorylated protein/loading controls. Values were obtained from duplicate measurements of two independent experiments and significant values are indicated; *P<0.05, in comparison to the stimulated control; ND, not detected. (C) Luciferase reporter assay. Relative luciferase activities (TOPFLASH/FOPFLASH) are shown as means±SD from three independent experiments after normalization against the values obtained with control cells (value = 1), and significant values are indicated; *P<0.05, in comparison to the control.

Several signaling molecules are important for the maintenance of mESC self-renewal and differentiation, e.g. LIF/STAT3, BMP/Smad, FGF/ERK and Wnt/β-catenin [9]–[12], [14], [16], [17], [23]. Therefore, we performed western blotting to determine whether the reduced sulfation affected the signal transduction. We observed a similar increase in the level of phosphorylated STAT3 in control and *PAPST*-KD cells after exposure to LIF (Figure 4A), which shows that sulfation is not required for LIF/STAT3 signaling. Depletion of CS chains by treatment with ChABC also had no effect on LIF/STAT3 signaling (Figure 4B). The levels of Smad1 phosphorylated in response to BMP4 and of ERK1/2 phosphorylated in response to basic FGF (bFGF) or FGF4 were reduced in *PAPST*-KD cells compared to those observed in control cells (Figure 4A). Depletion of CS chains by ChABC treatment had no effect on either BMP4 or FGF signaling (Figure 4B), whereas HS chains were involved in these signaling pathways in mESCs (Figure 4B and [17]). Defects in BMP4 and FGF signaling were also observed in *NDST1+2*-KD cells (Figure 4B). These results suggest that the reduction in BMP4 and FGF signaling in *PAPST*-KD cells was caused by reduced HS chain sulfation.

To date, LIF, Activin/Nodal and bFGF have been reported to contribute to mESC proliferation [7], [8], [46], [47]. It is well known that FGF signaling mediated by HS chains contributes to the proliferation of various types of cell [33]. Thus, we considered the possibility that autocrine/paracrine FGF signaling mediated by HS chains is involved in mESC proliferation. RT-PCR analysis showed that both R1 and E14TG2a cell lines expressed several *FGFs* and *FGF receptors* (*FGFRs*) (Figure S4A). Furthermore, the proliferation of mESCs treated with SU5402, an inhibitor of FGFR1 tyrosine phosphorylation, was reduced compared to that of control cells (Figure S4B), demonstrating that autocrine/paracrine FGF signaling mediated by FGFR1 contributes to mESC proliferation. Therefore, these results suggest that the reduced proliferation of *PAPST*-KD cells (Figure 3C) is due to a reduction in autocrine/paracrine FGF signaling, which in turn is caused by reduced HS chain sulfation (Figure 4A).

Previously, we reported that autocrine/paracrine Wnt/β-catenin signaling occurs in mESCs and that this signaling is regulated by HS chains [17]. We examined Wnt/β-catenin signaling using a luciferase reporter system and found a significant decrease in luciferase activity in both *PAPST1*-and *PAPST2*-KD cells compared to that in control cells (Figure 4C). Furthermore, we confirmed by western blotting that nuclear accumulation of β-catenin was reduced in both *PAPST1*-and *PAPST2*-KD cells compared to that in control cells (Figure S5). Depletion of CS chains by ChABC treatment did not affect luciferase activity in mESCs (Figure S3C). Depletion of HS chains using heparitinase reduced the amount of GSK3β that was phosphorylated in response to Wnt3a, whereas depletion of CS chains by ChABC treatment had no effect (Figure 4B). *NDST1+2*-KD cells exhibited a significant decrease in luciferase activity (Figure 4C). Thus, these results suggest that the reduction of Wnt/β-catenin signaling in *PAPST*-KD cells is caused by reduced HS chain sulfation.

Furthermore, the knockdown of both *PAPST1* and *PAPST2* had an additive effect on Wnt/β-catenin signaling, which suggested that the additive effect of *PAPST1* and *PAPST2* knockdown on self-renewal (Figure 3A and B) could be caused by this additional decrease in the level of signaling.

Taken together, the above results demonstrate that both PAPST1- and PAPST2-dependent sulfation regulates BMP/Smad, FGF/ERK and Wnt/β-catenin signaling in mESCs and suggest that the reduction in signaling is due to reduced sulfation of HS chains, not CS chains.

### The Reduction of Sulfation Induces Abnormal Differentiation into Three Germ Layers during EB Formation in mESCs

**Figure 5:**
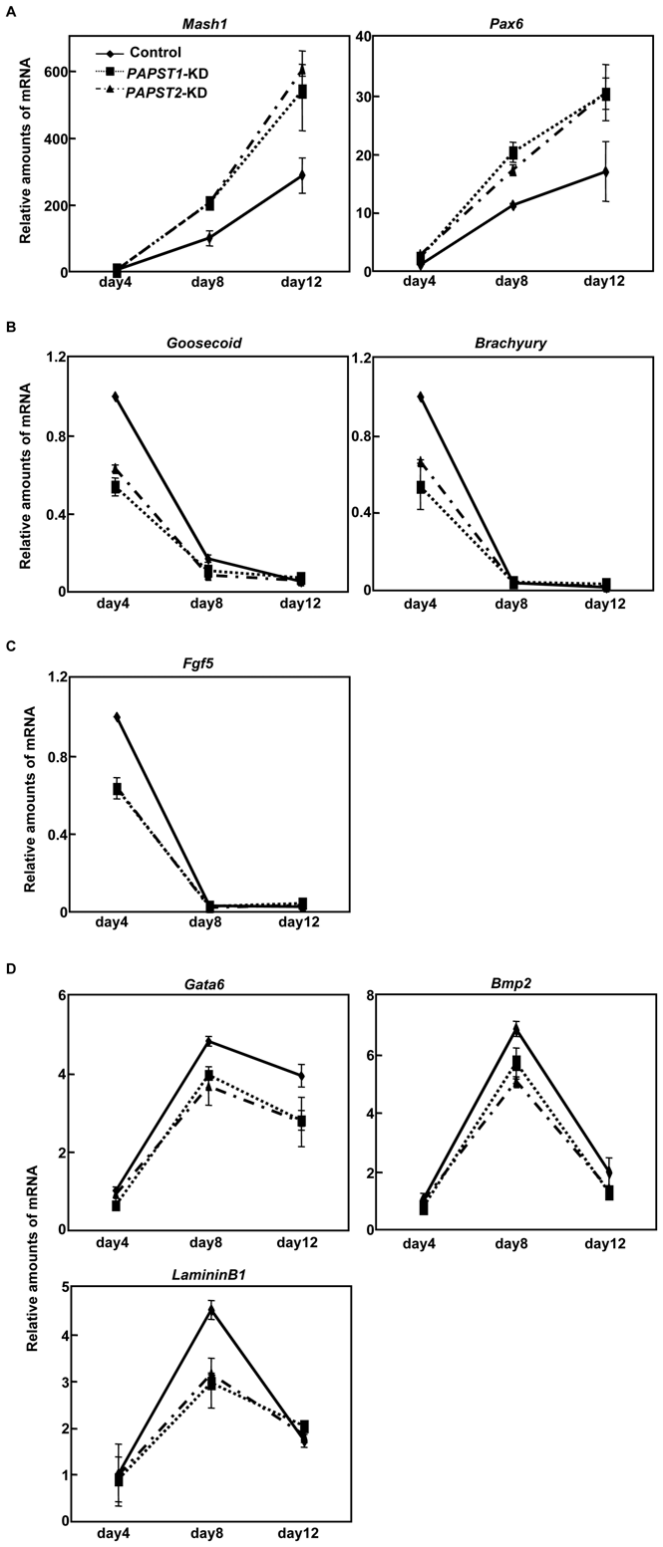
Abnormal differentiation was observed in *PAPST*-KD cells during EB formation. (A)–(D) Real time PCR analysis of germ layer markers 4, 8, and 12 days after EB formation (*A*, neurectoderm marker; *B*, mesoderm marker; *C*, primitive ectoderm marker; *D*, extraembryonic endoderm (ExE) marker). The results are shown after normalization against the values obtained with control EBs on day 4 (value = 1). The values shown are the means±SD of two independent experiments.

To determine further contribution of PAPST-dependent sulfation to differentiation of mESC, we examined the *in vitro* differentiation of *PAPST*-KD cells into EBs, which comprise the three germ layers, endoderm, mesoderm and ectoderm. To maintain the knockdown effects during long culture periods for EB formation, we used stable *PAPST*-KD cells. Control cells were stably transfected with *enhanced green fluorescent protein* (*EGFP*) siRNA expression vectors. Before EB formation, both *PAPST1*- and *PAPST2*-KD cells showed an approximately 50% reduction in *PAPST1* and *PAPST2* mRNA, respectively, as compared to control cells. Then we examined the expression of several germ layer markers by real time PCR 4, 8 and 12 days after EB formation (Figure 5). The expression of neurectoderm markers (*Mash1*, *Pax6*) increased in a time-dependent manner and the expression in *PAPST*-KD cells was higher than in control cells, indicating that neurectodermal differentiation was promoted in *PAPST*-KD cells. The expression of early mesoderm markers (*Brachyury*, *Goosecoid*) and a primitive ectoderm marker (*Fgf5*) decreased in a time-dependent manner and the expression in *PAPST*-KD cells was lower than in control cells, indicating that primitive ectodermal and mesodermal differentiations were inhibited in *PAPST*-KD cells. The expression of ExE lineage markers (*Gata6*, *LamininB1* and *Bmp2*) initially increased and reached a maximum level 8 days after EB formation, after which it decreased. The expression of these genes was lower in *PAPST*-KD cells than in control cells, indicating that endodermal differentiation was decreased in *PAPST*-KD cells. These results indicate that the *in vitro* differentiation of *PAPST*-KD cells is abnormal and that both PAPST1- and PAPST2-dependent sulfation contributes to differentiation of mESCs.

### The Reduction of Sulfation Promotes Neurogenesis

**Figure 6:**
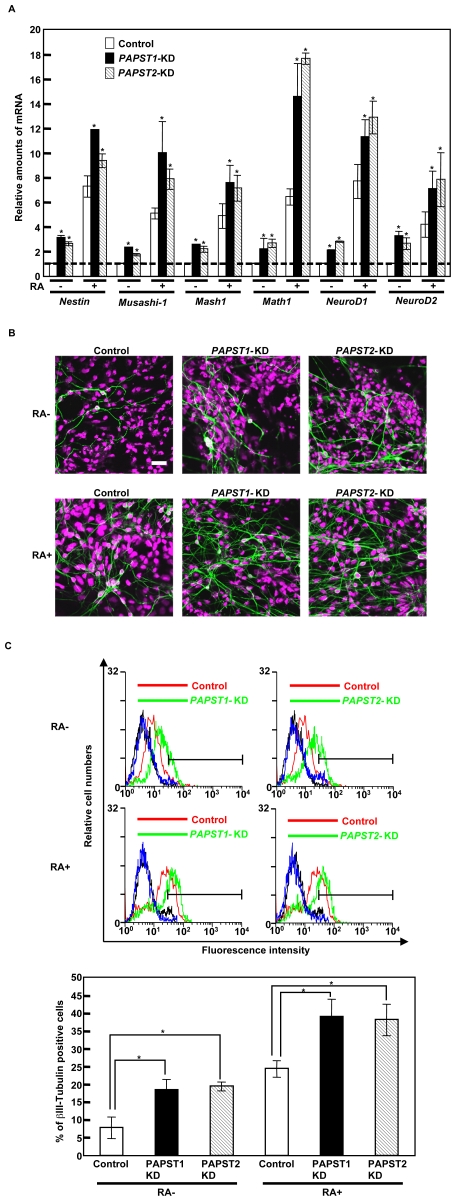
Neurogenesis was promoted in *PAPST*-KD cells. (A) Real time PCR analysis of neural differentiation markers 8 days after EB formation. The results are shown after normalization against the values obtained with control cells not treated with RA (value = 1). The values shown are the means±SD of duplicate measurements from two independent experiments and significant values are indicated; *P<0.05, in comparison to the control. (B) Immunocytochemical staining 6 days after replating of EBs. Representative confocal images from two independent experiments are shown. (βIII-Tubulin, green; PI, purple). Scale bar, 20 µm. (C) FACS analysis 6 days after replating of EBs using an anti-βIII-Tubulin antibody (black and blue lines represent the IgM isotype control for control and *PAPST*-KD cells, respectively). Three independent experiments were performed and representative results are shown. The histograms show the ratio of the mean fluorescent intensity within area of the insetted bar representing βIII-Tubulin positive cells to the mean fluorescent intensity over the total area±SD of three independent experiments and significant values are indicated; *P<0.01, in comparison to the control.

The results shown in Figure 5A indicated that *PAPST1* or *PAPST2* knockdown promoted the differentiation of mESCs into neurectoderm. Therefore, we investigated the neural differentiation of *PAPST*-KD cells. We examined the expression of neural differentiation markers by real time PCR 8 days after EB formation (Figure 6A). The expression of several neural markers, such as neural stem/progenitor cell markers (*Nestin*, *Musashi-1*) and proneural markers (*Mash1*, *Math1*, *NeuroD1* and *NeuroD2*), in *PAPST*-KD cells was higher than in control cells in both the presence and absence of all-trans retinoic acid (RA), indicating that the larger amounts of neural stem/neural progenitor cells and neural precursor cells existed in *PAPST*-KD cells. We examined further the ability of *PAPST*-KD cells to differentiate into neurons 6 days after replating EBs. Immunocytochemical staining showed that *PAPST*-KD cells appeared to generate dense networks of neurite outgrowths as compared to control cells in either the presence or the absence of RA (Figure 6B). We confirmed by western blotting that the level of βIII-Tubulin in *PAPST*-KD cells was quantitatively higher than that in control cells (Figure S6). Furthermore, FACS analysis showed that βIII-Tubulin positive cells were more abundant in *PAPST*-KD cells than in control cells (non-treated control, 7.7±3.0%; non-treated *PAPST1*-KD, 18.4±2.9%; non-treated *PAPST2*-KD, 19.4±1.2%; RA-treated control, 24.3±2.4%; RA-treated *PAPST1*-KD, 39.0±4.9%; RA-treated *PAPST2*-KD, 38.1±4.4%), which confirmed that differentiation into neurons was promoted in *PAPST*-KD cells (Figure 6C). These results demonstrate that both PAPST1- and PAPST2-dependent sulfation contributes to the neurogenesis of mESCs.

### PAPST-Dependent Sulfation of Both HS and CS Chains Regulates Several Signaling Pathways Required for the Correct Differentiation of mESCs during EB Formation

**Figure 7:**
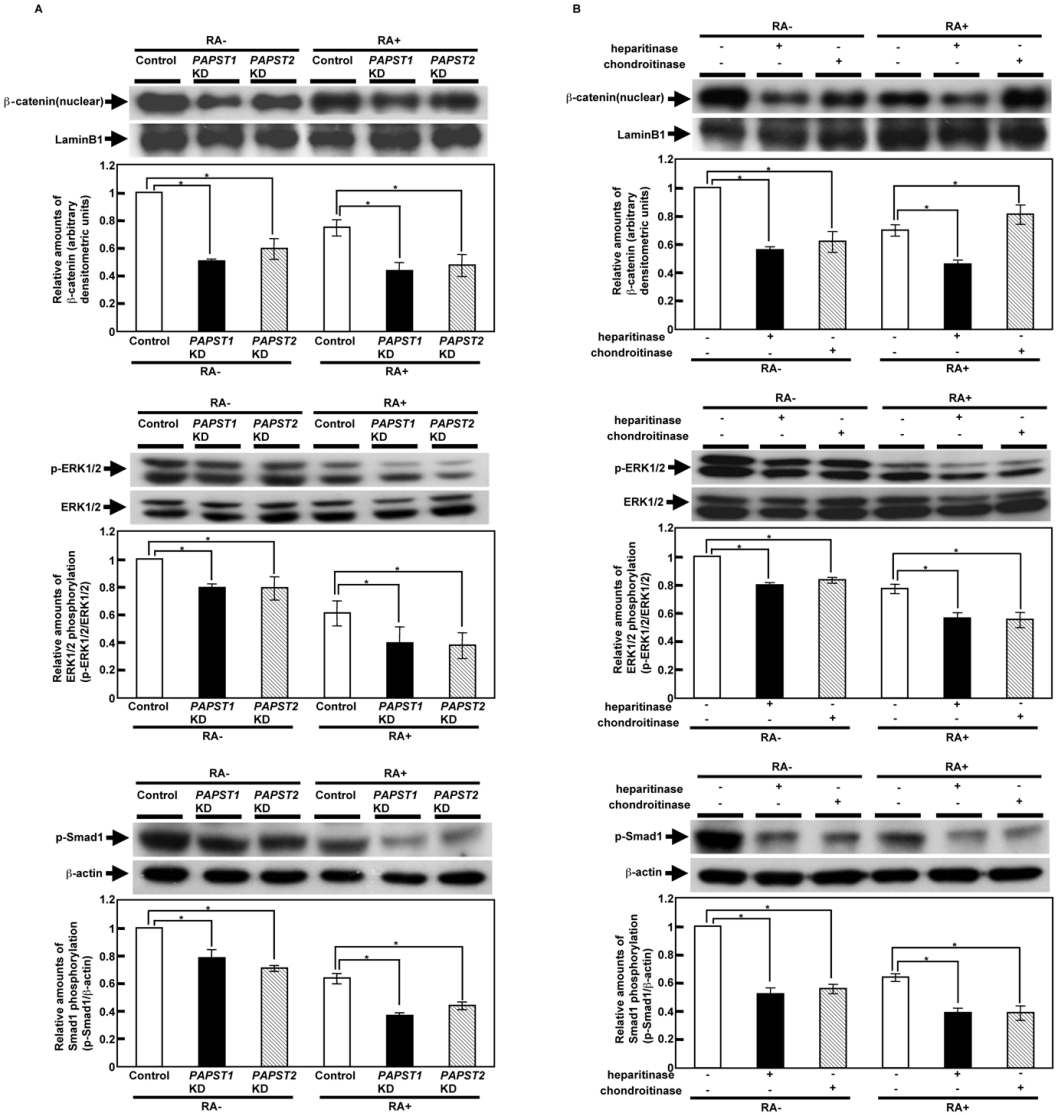
Signaling via a number of pathways was decreased in *PAPST*-KD cells during EB formation. (A) Western blot analysis of several signaling molecules in EBs on day 8. Two independent experiments were performed and representative results are shown. The histograms show mean densitometric readings±SD of β-catenin or the phosphorylated proteins/loading controls after normalization against the values obtained with control cells not treated with RA (value = 1). Values were obtained from duplicate measurements of two independent experiments and significant values are indicated; *P<0.01, in comparison to the control. (B) Western blot analysis of several signaling molecules in EBs on day 8 after heparitinase or chondroitinase treatment. Two independent experiments were performed and representative results are shown. The histograms show mean densitometric readings±SD of β-catenin or the phosphorylated proteins/loading controls after normalization against the values obtained with cells not treated with RA and enzyme (value = 1). Values were obtained from duplicate measurements of two independent experiments and significant values are indicated; *P<0.01, in comparison to cells not treated with enzyme.

Several signaling pathways, such as BMP, FGF and Wnt, play important roles in the mouse embryo during early embryogenesis and mESC differentiation [20]. Thus, we examined whether defects in these signaling pathways contribute to the abnormal differentiation of *PAPST*-KD EBs, especially the promotion of neurogenesis. We performed western blotting of BMP, FGF and Wnt signaling molecules in control and *PAPST*-KD cells 8 days after EB formation. As shown in Figure 7A, the nuclear accumulation of β-catenin and the levels of phosphorylated ERK1/2 and Smad1 were reduced in *PAPST*-KD cells as compared to control cells, which indicated that Wnt/β-catenin, FGF/ERK and BMP/Smad signaling were reduced in *PAPST*-KD EBs. Furthermore, Wnt/β-catenin, FGF/ERK and BMP/Smad signaling were reduced in EBs depleted for HS and CS chains in the absence of RA (Figure 7B). In EBs treated with RA, HS chain depletion reduced signaling via all these pathways as compared with untreated EBs. In contrast, CS chain depletion reduced FGF/ERK and BMP/Smad signaling to a similar extent as HS chain depletion but promoted Wnt/β-catenin signaling (Figure 7B).

To date, *K_D_* values have been determined for the binding of FGFs (e.g., bFGF and FGF4) to HS chains and to CS-E (GlcAβ1–3GalNAc(4,6-SO_3_)), a particular form of CS chain [48], [49]. Although the binding of BMP4 to HS chains has been demonstrated [50], the *K_D_* value has not been determined. In addition, the binding of BMP4 to CS chains has not been demonstrated. Therefore, we performed surface plasmon resonance (SPR) analysis for Wnt3a and BMP4 against heparin, a structural analogue of HS chains, and CS-E. BMP4 bound to both heparin and CS-E (*K_D_* = 69.4 nM and 30.0 nM, respectively) (Table 1). Wnt3a also bound to both heparin and CS-E, as described in other recent reports [51] (*K_D_* = 26.0 nM and 27.3 nM, respectively) (Table 1). Thus, it was clearly shown that the sulfate groups of HS and CS chains contribute to the binding of Wnt3a and BMP4 to both HS and CS chains.

**Table 1:** The apparent association (*k_a_*), dissociation (*k_d_*) rate constants and equilibrium dissociation constants (*K_D_*) for the interaction of BMP4 and Wnt3a with immobilized heparin or CS-E.

Ligand	GAG	*k_a_* (M^−1^Sec^−1^)	*k_d_* (Sec^−1^)	*K_D_* (nM)
BMP4	Heparin	2.76×10^5^	1.92×10^−2^	69.4
BMP4	CS-E	1.44×10^5^	4.33×10^−3^	30.0
Wnt3a	Heparin	2.22×10^5^ a	5.77×10^−3^ a	26.0 a
Wnt3a	CS-E	8.26×10^5^	2.26×10^−2^	27.3

The *k_a_*, *k_d_* and *K_D_* values were determined by SPR analysis.

a The *k_a_*, *k_d_* and *K_D_* values of Wnt3a against heparin have been published in our previous paper [17].

These results demonstrate that both PAPST1- and PAPST2-dependent sulfation regulates BMP/Smad, FGF/ERK and Wnt/β-catenin signaling during EB formation and indicate that this regulation is presumably dependent on both HS and CS chains. In addition, the results demonstrate that the reduction in signaling contributes to the abnormal differentiation of *PAPST*-KD cells, such as promotion of neurogenesis.

## Discussion

**Figure 8:**
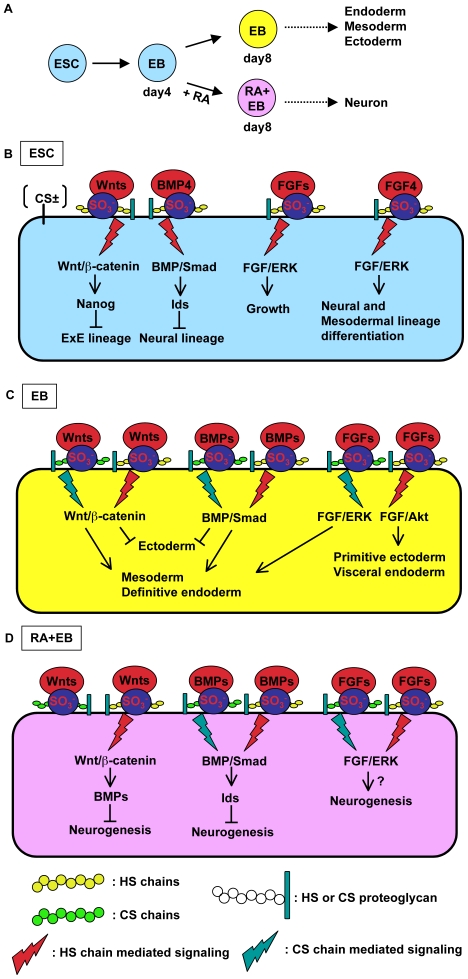
PAPST-dependent sulfation contributes to mESCs in both undifferentiated and differentiated state. (A) *In vitro* differentiation flowchart of mESCs. EBs that are not treated with RA produce cells from all three germ layers (endoderm, mesoderm and ectoderm) whereas RA-treated EBs produce neurons after further adherent culture. (B) PAPST-dependent sulfation of HS chains regulates the extrinsic signaling (by BMP, FGF and Wnt) that is required for the growth and pluripotency of mESCs. In undifferentiated mESCs, the transduction of extrinsic signals is dependent on the sulfation of HS chains, but not CS chains, and this maintains growth and pluripotency. As shown in this study, autocrine/paracrine FGF signaling contributes to the growth of mESCs. In particular, FGF4 signaling contributes to the differentiation state of the mESCs [23]. (C) PAPST-dependent sulfation of both HS and CS chains regulates the extrinsic signaling (by BMP, FGF and Wnt) that is required for normal differentiation of EBs. During EB differentiation into the three germ layers, the transduction of the extrinsic signals is dependent on the sulfation of both HS and CS chains. Wnt and BMP signaling inhibit ectodermal differentiation and contribute to mesodermal and definitive endodermal differentiation [20]–[22], [24], [25], [63]. FGF/ERK and FGF/Akt signaling contribute to mesodermal and definitive endodermal differentiation and primitive ectodermal and visceral endodermal differentiation, respectively [20], [61], [62]. (D) PAPST-dependent sulfation of HS and CS chains regulates the extrinsic signaling (by BMP, Wnt and FGF) that is required for neuronal differentiation of RA-treated EBs. During RA-treated EB differentiation, the transduction of extrinsic signals is dependent on the sulfation of both HS and CS chains and results in neuronal differentiation. Wnt and BMP signaling inhibit neurogenesis [24], [25] and FGF (e.g., bFGF) signaling may contribute to neurogenesis. CS chains regulate Wnt signaling negatively, presumably by sequestering Wnt proteins and preventing them interacting with Wnt receptors.

Until now, the functional roles of sulfation during early embryogenesis and in ESCs have not been described well. Here, we demonstrate that both PAPST1- and PAPST2-dependent sulfation is important for extrinsic signaling pathways, such as BMP/Smad, FGF/ERK and Wnt/β-catenin, in both undifferentiated and differentiated mESCs. In the undifferentiated state, sulfation of HS chains contributes mainly to the maintenance of mESCs. During the differentiation of mESCs, namely during EB formation, sulfation of both HS and CS chains contributes predominantly to the normal differentiation of EBs (Figure 8).

Sulfation is an essential modification of carbohydrates and proteins. As shown in Figure 4 and 7, various types of signaling were reduced in *PAPST*-KD cells, presumably due to the reduced sulfation of specific carbohydrates and proteins. Two sulfated carbohydrates, HS and CS, were found on the surface of mESCs and EBs ([17] and Figure S7A). We also examined the expression of other sulfated carbohydrates. In mESCs, 3′-sulfo-Le^a^, HNK-1 carbohydrate, and sulfatide SM4 were not expressed. The expression level of sulfatide SM3 was low and the reduction of SM3 sulfation in *PAPST*-KD cells was slight. Therefore, we assume that the reduction of SM3 sulfation in the *PAPST*-KD cells is not responsible for the effects observed in this study. However, further studies will be required to elucidate the function of SM3 in mESCs. In EBs, HNK-1 carbohydrate was detected at appreciable levels after induction of neural differentiation by treatment with RA (Figure S7B), whereas 3′-sulfo-Le^a^ and sulfatides SM3 and SM4 were not detected by FACS analyses in either RA-treated or non-treated EBs (Data not shown). HNK-1 carbohydrate is expressed in the nervous system, including in neural precursor cells [52], [53]; however brain development is generally normal in mice in which glucuronyltransferase, which is required for HNK-1 carbohydrate synthesis, has been mutated [54]. Therefore, the contribution of HNK-1 carbohydrate to the neural differentiation of EBs upon RA treatment is considered minor. As mentioned above, the sulfated carbohydrates 3′-sulfo-Le^a^, HNK-1 carbohydrate and sulfatides SM3 and SM4 are assumed not to have an important functional role in mESCs and EBs.

The other candidates for sulfation are tyrosine residues on proteins. The sulfation of proteins occurs on specific tyrosine residues as they enter the secretory pathway. However, according to sulfation consensus prediction algorithms (e.g. The Sulfinator) [55], the relevant extrinsic factors, such as Wnt3a, BMP2, BMP4 and several FGFs, are not predicted to be substrates for tyrosine sulfation. This suggests that the reduction in signaling via these pathways in *PAPST*-KD cells is not due to reduced tyrosine sulfation of extrinsic factors. On the other hand, specific sulfated regions of HS and CS chains are required for the binding of extrinsic factors and subsequent signal transduction [41], [42], [51], [56]. Indeed, HS and CS chains contribute to extrinsic factor binding and several signaling pathways in both ESCs and EBs or in EBs, respectively ([17], Figure 4 and 7B and Table 1). These results suggest that the reduction in extrinsic signaling in *PAPST*-KD cells is mainly due to a reduction in HS chain sulfation in ESCs and a reduction in both HS and CS chain sulfation in EBs (Figure 8).

As shown in Fig. 2B, a 30–40% reduction in HS and CS chain sulfation was observed, whereas PAPST activity was reduced by approximately 20%. These substantial effects on HS and CS chain sulfation despite the modest reduction in PAPST activity may be explained as follows. Sulfations of HS and CS chains are modified by several different sulfotransferases. NDST is responsible for the first step of HS chain sulfation and has a key role in determining the sulfated structures of HS chains. The *K_m_* value of NDST1 for PAPS is 40.7 µM [57], whereas PAPST1 and PAPST2 showed relatively low apparent *K_m_* values for PAPS (1.54 µM and 1.49 µM, respectively). Thus, the effect of PAPST1 and PAPST2 can be considered to be significant, because the *K_m_* values of PAPST1 and PAPST2 for PAPS are lower than the *K_m_* value of the sulfotransferase. Therefore, taking into account this assumption, the reduction in HS and CS sulfation can be substantial despite the limited reduction of PAPST activity.

Until recently, it was considered that the pluripotency of mESCs in adherent culture is maintained by a balance among extrinsic signaling pathways, such as LIF, BMP and FGF signaling, and also by a combination of extrinsic and intrinsic factors, such as Oct3/4 and Nanog [4]–[6], [58]. However, we have reported that autocrine/paracrine Wnt/β-catenin signaling through HS chains contributes to the inhibition of mESC differentiation into the ExE lineage by maintaining Nanog expression [17]. In this earlier study, we proposed that proper control of Wnt signaling, in addition to BMP, FGF and LIF signaling, is required for the maintenance of mESC pluripotency. In the present study, we demonstrated that BMP, FGF and Wnt signaling were regulated by the sulfation of HS chains (Figure 8B). Signaling by BMP4, FGFs and Wnts, but not LIF, was disrupted in *PAPST*-KD cells that differentiated spontaneously into the ExE lineage in adherent culture (Figure 4 and S2). Reduction of Wnt signaling induces differentiation into the ExE lineage due to a reduction in the level of Nanog. As reported previously, the level of Oct3/4 expression affects both mESC differentiation and lineage choice [59]. An increase in expression of Oct3/4 of less than twofold causes mESCs to differentiate into primitive endoderm and mesoderm. In contrast, repression of Oct3/4 induces loss of pluripotency and dedifferentiation to trophectoderm. However, as shown in Figure S2, *PAPST*-KD cells differentiated into the ExE lineage, but not into mesoderm or trophectoderm. Hence, the lineage choice of *PAPST*-KD cells may be affected mainly by the reduction in Nanog that occurs due to reduced Wnt signaling rather than by Oct3/4. Furthermore, reduction of FGF4/ERK signaling inhibits differentiation into neural and mesodermal lineages and subsequently induces differentiation into other lineages such as ExE, whereas reduction of BMP signaling induces the neural differentiation of mESCs [23], [58], [60]. Thus, the spontaneous differentiation of *PAPST*-KD cells into ExE lineage cells is presumably due to a synergetic effect from reduced Wnt and FGF signaling due to reduced HS chain sulfation.

In EBs, signaling by BMP, FGF or Wnt was regulated by sulfation of both HS and CS chains and sulfation of these GAG chains regulated the differentiation of EBs (Figure 5, 7B and 8C). As shown in Figure 5C and D, differentiation into ExE lineage cells (including visceral endoderm) and primitive ectoderm was reduced in *PAPST*-KD cells. It has been reported that FGF signaling is required for the normal differentiation of EBs, differentiation of visceral endoderm, and subsequent differentiation of primitive ectoderm [61]. This report supports our proposal that defects in the differentiation of the visceral endoderm and primitive ectoderm in *PAPST*-KD cells resulted from a reduction in FGF signaling (Figure 8C). Unlike the control cells, *PAPST*-KD cells failed to differentiate into mesodermal cells during EB formation (Figure 5B), presumably due to defects in BMP, FGF and Wnt signaling that were caused by the reduced sulfation of both HS and CS chains (Figure 7 and 8C). This notion is supported by the finding that mesodermal differentiation is induced by BMP, FGF and Wnt signaling in mouse embryos and ESCs [20], [62].

Sulfation of both HS and CS chains contributed to the decision between ectodermal and mesodermal fates by regulating BMP and Wnt signaling (Figure 8C). It is known that signaling by both BMP and Wnt is essential for this decision [21], [22], [63]. The reduction of BMP and Wnt signaling inhibits mesodermal differentiation and enhances ectodermal differentiation. As shown in Figure 5A and 7A, during EB formation, ectodermal differentiation was promoted in *PAPST*-KD cells and both BMP and Wnt signaling were reduced, which demonstrated that the adoption of an ectodermal fate was enhanced in *PAPST*-KD cells, mainly due to the reduction of these two types of signaling.

Furthermore, sulfation of both HS and CS chains regulated BMP, FGF and Wnt signaling during neural differentiation of EBs after RA treatment. Decreased signaling in *PAPST*-KD cells promoted both the production of neural precursor cells and further neural differentiation (Figure 6, 7A and 8D). Neural specification, which can be achieved by the formation of ESC spheres, has been shown to require signaling by endogenous FGF (e.g. bFGF). FGF signaling that is regulated by the sulfation of both HS and CS chains may also contribute to neurogenesis in RA-treated EBs (Figure 8D). On the other hand, previous reports have demonstrated that signaling by BMP and Wnt inhibits the neurogenesis of mESCs via EB formation [24], [25]. As described above, neurogenesis was promoted in *PAPST*-KD cells. Taken together, these results suggest that the reduction of BMP and Wnt signaling mainly affects the promotion of neurogenesis.

As shown in Figure 7B and 8D, HS chains regulate Wnt signaling positively in RA-treated EBs, whereas CS chains regulate the signaling negatively. However, Wnt signaling was reduced overall in RA-treated *PAPST*-KD cells during EB formation (Figure 7A), which indicated that the contribution of HS chains predominated. CS chains have been proposed to function in two distinct ways: they can interact with a ligand either to present it to its receptor (positive function) or to sequester it from its receptor (negative function) [64]. However, the factors that distinguish these two functions have not yet been clarified. It has been demonstrated that the binding of CS chains to ligands, including heparin binding growth-associated molecules, is regulated by the length of the chains and the nature of the sulfated structures [65], which change during development and vary among different tissues and core proteins. With respect to Wnt signaling, CS chains exhibited a negative function in EBs treated with RA but a positive function in untreated EBs, as shown in Figure 7B. These different effects of CS chains on Wnt signaling may depend on the length and sulfated structures of the CS chains, including the core proteins. Furthermore, it has been demonstrated that neural stem/progenitor cells depleted for CS chains are impaired in neuronal differentiation [66], but the underlying mechanisms have not been elucidated. As mentioned above, Wnt signaling contributes to the inhibition of neural differentiation. Thus, we propose that CS chains promote the differentiation of neural stem/progenitor cells into the neuronal lineage by sequestering Wnt proteins away from their receptors and inhibiting Wnt signaling (Figure 8D).

Johnson et al. [39] have reported recently that HS-null mESCs show no defects in pluripotency, e.g. there is no reduction in *Oct3/4* expression, but neural differentiation in these cells is disrupted due to defects in FGF4 signaling. Their results differ from ours in certain aspects. 1) A previous report [37] had shown that, in HS-null mESCs, the amount of CS chains is increased, whereas in HS-KD mESCs, this increase is not observed, as had been described previously [17]. In HS-null mESCs, the additional CS chains might contribute to different signaling pathways and compensate for the function of HS chains. 2) Their protocol for neural differentiation was different from ours. They used a Sox1-EGFP reporter cell line in adherent culture: under these culture conditions neural differentiation is induced by autocrine FGF4 signaling [60]. On the other hand, we used EB formation plus RA treatment: under these conditions autocrine signaling by BMP and Wnt inhibits neural differentiation [24], [25]. Thus, the different results with respect to neural differentiation could be caused by the use of different culture conditions that induce neural differentiation by different signaling pathways.

The sulfation patterns of HS chains are tissue specific and mutations in enzymes that are involved in HS chain sulfation lead to severe developmental abnormalities [36], [38], [43]. Thus, control of HS chain structure is essential for the spatiotemporal regulation of cellular differentiation and growth throughout development. However, the patterning of HS chain modification during development has not been well characterized. Recently, it has been demonstrated that the concentration of PAPS influences the patterning of HS chain sulfation in a cooperative manner with NDST [67]. We propose that PAPSTs play important roles in regulating the sulfation levels and patterning of CS chains, as well as HS chains and other sulfated substrates, in a developmental context and contribute to several signaling pathways that are required for normal development.

## Materials and Methods

### Materials

GDP-[2-^3^H]mannose (40 Ci/mmol), UDP-[1-^3^H]glucose (20 Ci/mmol), UDP-*N*-acetyl [6-^3^H]D-galactosamine (20 Ci/mmol), UDP-*N*-acetyl [6-^3^H(N)]D-glucosamine (60 Ci/mmol), UDP-[^14^C(U)]glucuronic acid (300 mCi/mmol), UDP-[^14^C(U)]xylose (264 mCi/mmol), UDP-[6-^3^H]galactose (20 Ci/mmol), and carrier-free [^35^S]Na_2_SO_4_ (100 mCi/ml) were purchased from American Radiolabeled Chemicals Inc. GDP-[2-^3^H]fucose (17.5 Ci/mmol), CMP-[9-^3^H]sialic acid (33.6 Ci/mmol), and [^35^S]PAPS (1.66 Ci/mmol) were purchased from Perkin Elmer Life Sciences Inc. Zymolyase 100T was obtained from Seikagaku Corp. All the other reagents used in this study were of the highest purity grade available commercially.

### Cell Culture and Transfection

R1 [68] and E14TG2a [69] mESC lines were maintained on mouse embryonic fibroblasts (MEFs) inactivated with 10 µg/ml mitomycin C (Sigma) in ESC medium (DMEM supplemented with 15% FBS {Hyclone}, 1% penicillin/streptomycin {Gibco}, 0.1 mM 2-mercaptoethanol {Gibco}, and 0.1 mM non-essential amino acids {Gibco}) with 1000 U/ml LIF (Chemicon). We generated siRNA expression plasmids that targeted *PAPST1*, *PAPST2*, *NDST1*, *NDST2*, and *EGFP*, a negative control, by inserting the appropriate dsDNAs between the BamHI and HindIII sites of pSilencer 3.1-H1 (Ambion) or pSUPER.retro.puro (OligoEngine). The siRNA sequences used for RNAi were designed as described previously [70] using “siDirect”, which is based on accelerated off-target search algorithm [71] and are listed in Table S1. We designed two kinds of constructs, *PAPST1-1* and *-2*, *PAPST2-1* and *-2*, *NDST1-1* and *-2*, and *NDST2-1* and *-2*, targeting *PAPST1*, *PAPST2*, *NDST1*, and *NDST2*, respectively. We describe mESCs that have been transfected with *EGFP* siRNA expression vectors as “control cells” throughout this paper.

For transient knockdown of *PAPST* or *NDST* mRNA by RNAi, siRNA expression plasmids for *PAPST* or *NDST* were transfected into mESCs as follows. Prior to transfection, the mESCs were harvested, replated at 1×10^6^ cells on gelatin-coated feeder-free 60 mm tissue culture dishes (Iwaki) in ESC medium with LIF, and incubated for 16 h. On day 1, the cells were transfected with an siRNA expression plasmid (2 µg per culture dish) using Lipofectamine 2000 (Invitrogen). On day 2, the cells were harvested and replated at 3×10^6^ cells on gelatin-coated feeder-free 60 mm tissue culture dishes in ESC medium with LIF and 2 µg/ml puromycin (Sigma). In general, puromycin selection of transfected cells was carried out for 24 h. Transfection efficiency was approximately 60%, but only transfected cells survived after puromycin selection. On day 3 (two days after transfection), the transfected cells were harvested and analyzed as described below.

Stable knockdown of *PAPST* mRNA was carried out as follows. To produce retrovirus, the pSUPER.retro.puro constructs were transfected into ecotropic virus-packaging (PLAT-E) cells. Virus-containing supernatants derived from these PLAT-E cultures were mixed with 8 µg/ml polybrene (Sigma) and mESCs were incubated with the virus/polybrene mixtures for 24 h. After infection, the cells were replated with ESC medium containing LIF and 2 µg/ml puromycin and cultured for 5–7 days.

For EB formation, the cells were transferred to Low Cell Binding 60 mm dishes (Nunc) and cultured in ESC medium without LIF. For neuronal differentiation, 1 µM RA (Sigma) was added on day 4 and day 6 after EB formation [72]. On day 8, 200 EBs were plated onto PDL/laminin-coated 60 mm dishes (Becton Dickinson) in DMEM-F12 containing N2 supplement (Gibco). The medium was replaced every other day and the cells were incubated for 6 days.

### Metabolic Labeling

To measure total sulfate incorporation into cellular proteins, 3×10^6^ mESCs were replated 2 days after transfection on gelatin-coated 60 mm dishes and incubated in sulfate-free ESC medium with LIF, puromycin and 100 µCi/ml [^35^S]Na_2_SO_4_. After labeling for 24 h, the cells were washed with Phosphate Buffered Saline (PBS) and lysed with lysis buffer (50 mM Tris-HCl pH 7.4, 150 mM NaCl, 1% Triton X-100, protease inhibitors). Fifty micrograms of protein were precipitated with 10% trichloroacetic acid and washed with 5% trichloroacetic acid, followed by cold acetone. The precipitate was dried, dissolved in 0.5 N NaOH and the amount of radioactivity present was quantitated using a scintillation counter.

To measure sulfate incorporation into cell surface HS and CS chains, after labeling, the cells were washed twice with PBS and then treated with 1 mg/ml trypsin (WAKO) for 10 min at 37°C. The trypsin was neutralized with 2 mg/ml trypsin inhibitor (Roche). After centrifugation, the supernatants were treated with 0.5 M NaOH at 4°C overnight and neutralized with 1 M acetic acid. The cell pellets were used for normalization as described below. The labeled *O*-linked glycans were desalted in a PD-10 column (GE Healthcare) and GAG chains were isolated by anion exchange chromatography on HiTrap DEAE FF (GE Healthcare) using sodium phosphate buffer (pH 6.0) containing 1.0 M NaCl as the eluent. After desalting, GAG chains were incubated in the presence of 5 mU heparitinase I and II (Seikagaku Corp.) or 100 mU/ml ChABC (Seikagaku Corp.) at 37°C overnight. The lyase products of HS or CS were recovered with Microcon YM-3 ultrafiltration devices (*M*r 3,000 cut-off; Millipore). The amount of radioactivity present was quantified using a scintillation counter. For normalization of the radioactivity with total amount of protein, the cells pellets obtained as above were lysed with lysis buffer and then the protein was quantified.

To measure molecular size of HS and CS chains, preparation and measurement of ^35^S-labeled HS and CS chains were performed as described previously [17].

### Isolation of Mouse PAPS Transporter cDNA and Construction of Expression Plasmids

The mouse *PAPST1* and *PAPST2* genes were identified and cloned using the same procedures as described previously [31]. To obtain the cDNA of NM_028662 and NM_134060, mouse genes that were identified in this study, and to create recombination sites for the GATEWAY™ cloning system (Invitrogen), we used two steps of *attB* adaptor PCR and prepared *attB*-flanked PCR products. The first gene-specific amplification was performed using Platinum® Pfx DNA polymerase (Invitrogen), a cDNA from mESCs and the following primers: *PAPST1*, forward primer with *attB1*, 5′-AAAAAGCAGGCTTCGCCGCCACCATGGACGCCAGATGGTGG-3′ and a reverse primer with *attB2*, 5′-AGAAAGCTGGGTTCACCTTCTGTACTGGGGG-3′; *PAPST2*, forward primer with *attB1*, 5′-AAAAAGCAGGCTTCGCCGCCACCATGGACCTCAAGTTCAACAAC-3′ and a reverse primer with *attB2*, 5′-AGAAAGCTGGGTTCACAGTCTGTGCCAACGTC-3′. The insertion of a complete *attB* adaptor and cloning into the pDONRTM201 vector were performed in accordance with the manufacturer's protocol to create an entry clone for use during the subsequent subcloning steps. The entry clone was subcloned into a yeast expression vector, YEp352GAP-II [73], by using the GATEWAY™ cloning system in accordance with the manufacturer's protocol. A 3 x influenza HA epitope tag was inserted into the expression vectors at the position corresponding to the C terminus of the expressing protein.

### Subcellular Fractionation of Yeast and Transport Assay

Yeast (*Saccharomyces cerevisiae*) strain W303-1a (*MATa*, *ade2-1*, *ura3-1*, *his3-11*,*15*, *trp1-1*, *leu2-3*,*112*, and *can1-100*) was transformed by the lithium acetate procedure using YEp352GAP-II inserted with HA-tagged *PAPST1* or *PAPST2*. These transformed yeast cells were grown at 25°C in a synthetic defined medium, which did not contain uracil, for selecting transformants. Subcellular fractionation and nucleotide sugar transport assays were performed as described previously [31]. The cells were harvested, washed with ice-cold 10 mM NaN_3_, and converted into spheroplasts by incubation at 37°C for 20 min in spheroplast buffer (1.4 M sorbitol, 50 mM potassium phosphate pH 7.5, 10 mM NaN_3_, 40 mM 2-mercaptoethanol, and 1 mg of Zymolyase 100T/g of cells). The spheroplasts were pelleted using a refrigerated centrifuge and washed twice with 1.0 M ice-cold sorbitol to remove traces of zymolyase. The cells were suspended in ice-cold lysis buffer (0.8 M sorbitol in 10 mM triethanolamine pH 7.2, 5 µg/ml of pepstatin A, and 1 mM phenylmethylsulfonyl fluoride) and subsequently homogenized using a Dounce homogenizer. The lysate was centrifuged at 1,000×*g* for 10 min to remove the unlysed cells and cell wall debris. The supernatant was then centrifuged at 10,000×*g* for 15 min at 4°C, which yielded a pellet of P10 membrane fraction. The supernatant was further centrifuged at 100,000×*g* to yield a pellet of P100 Golgi-rich membrane fraction. Each Golgi-rich membrane fraction (100 µg of protein) was then incubated in 50 µl of reaction buffer (20 mM Tris-HCl pH 7.5, 0.25 M sucrose, 5.0 mM MgCl_2_, 1.0 mM MnCl_2_, and 10 mM 2-mercaptoethanol) that contained 1 µM radiolabeled substrate at 32°C for 5 min. After incubation, the radioactivity incorporated in the microsomes was trapped using a 0.45-µm nitrocellulose filter (Advantec MFS) and measured using liquid scintillation. The amount of incorporated radioactivity was calculated as the difference from the background value obtained from the same assay for 0 min for each sample.

### Measurement of PAPS Transport in mESC

Two days after transfection, cells were harvested and subcellular fractionation and measurement of PAPS transport was performed like as described above. Each Golgi-rich membrane fraction (50 µg of protein) was incubated in 100 µl of reaction buffer that contained 1 µM [^35^S] PAPS.

### FACS Analysis

FACS analysis was performed 3 days after transfection or 8 days after EB formation. Cells were harvested and the cell suspension was incubated with primary antibodies diluted in FACS buffer (0.5% bovine serum albumin (BSA) and 0.1% sodium azide in PBS). After washing, the cell suspension was incubated with FITC-conjugated secondary antibody (Sigma) diluted in FACS buffer. Cell sorting and analysis were performed using a FACSAria Cell Sorter (Becton Dickinson). We used the following as primary antibodies: mouse IgM isotype control (Chemicon), the anti-3′-sulfo-Le^a^ antibody 91.9H [74], an anti-HNK-1 carbohydrate antibody (Becton Dickinson), the anti-HS antibody 10E4 (Seikagaku Corp.), the anti-HS antibody HepSS-1 (Seikagaku Corp.), the anti-CS antibody 2H6 (Seikagaku Corp.), the anti-SM3 antibody 49-D6 (Seikagaku Corp.) and the anti-SM4 antibody O4 (Chemicon).

Detection of βIII-Tubulin positive cells was performed as follows. Six days after neuronal differentiation, cells were harvested, washed and stained with Propidium Iodide (PI) (Becton Dickinson) to allow the elimination of dead cells by gating. After washing, the cells were fixed with 2% paraformaldehyde and permeabilized with 0.5% saponin. Staining with the primary antibody (anti-βIII-Tubulin antibody; Chemicon) and secondary antibody (FITC-conjugated anti-mouse IgG; Chemicon) was performed in PBS containing 0.1% saponin.

### Measurement of Proliferation

Two days after transfection, cells were harvested and replated in triplicate at 0.8×10^4^ cells per well in 96-well 0.2% gelatin-coated plates in ESC medium with LIF. Cell counting kit-8 (Dojindo) was added after 48 h and incubated further for 2 h. The soluble formazan product was measured at 450 nm.

To examine the involvement of autocrine/paracrine FGF signaling in mESC proliferation, we treated mESCs with 10 µM SU5402 (Calbiochem) during culture.

### Measurement of Self-Renewal

Two days after transfection, cells were harvested and replated at 1×10^4^ cells per gelatin-coated 60 mm tissue culture dish in ESC medium with LIF. For detection of undifferentiated cells, cells were fixed and stained with BCIP-NBT (Nacalai Tesque) 5 days after replating. Alkaline phosphatase (AP) positive colonies were counted by microscopic examination. Colonies of tightly packed and flattened AP positive cells were counted as undifferentiated, and colonies of mixtures of unstained and stained cells and entirely unstained cells with flattened irregular morphology were considered differentiated.

### Analysis of Proteins by Immunoblotting

Three days after transfection, the mESC culture solution was replaced with serum-free ESC medium without LIF for 4 h and the cells were stimulated for 20 min with 1000 U/ml LIF, 10 ng/ml BMP4 (R&D Systems) or 100 ng/ml Wnt3a (R&D Systems), or for 5 min with 40 ng/ml bFGF (Upstate Biotechnology) or 10 ng/ml FGF4 (R&D Systems). To deplete HS or CS chains, mESCs were incubated in the presence of 5 mU heparitinase I and II or 100 mU/ml ChABC for 2 h before stimulation with extrinsic factors. We confirmed the reduction of HS or CS structure by FACS analysis (Figure S8). Cells were lysed with lysis buffer (50 mM Tris-HCl pH 7.4, 150 mM NaCl, 1% Triton X-100, 1 mM Na_3_VO_4_, 10 mM NaF, protease inhibitors). Isolation of nuclear extracts was performed as described previously [17]. To deplete HS or CS chains during EB formation, EBs were incubated in the presence of 5 mU heparitinase I and II or 100 mU/ml ChABC, respectively, for 4 days. We confirmed the reduction of HS or CS structure by FACS analysis (Data not shown). For analysis of HA-tagged PAPST1 or PAPST2, protein from each sample was added to 3 x SDS sample buffer (New England Biolabs Inc.) and subsequently incubated at 4°C for 12 h.

Samples containing 5 µg of cell lysate or nuclear extract were separated by 10% SDS-PAGE and transferred onto PVDF membranes (Millipore). After blocking, the membranes were incubated with antibodies against STAT3 (Becton Dickinson), phosphorylated STAT3 (Tyr705; Becton Dickinson), ERK1/2 (Cell Signaling Technology), phosphorylated ERK1/2 (Thr183 and Thr185; Sigma), phosphorylated Smad1 (Ser463 and Ser465; Cell Signaling Technology), β-actin (Sigma), β-catenin (Cell Signaling Technology), Lamin B_1_ (Zymed), βIII-Tubulin or HA (Roche). The membranes were then incubated with the appropriate peroxidase-conjugated secondary antibodies (Cell Signaling Technology), washed and developed with ECL Plus reagents (GE Healthcare).

### Measurement of Luciferase Reporter Activity

Transactivation of β-catenin on T-cell-specific factor (Tcf) was determined with a luciferase reporter assay. siRNA expression plasmid (2 µg) was cotransfected with reporter plasmid such as, TOPFLASH (2 µg, containing three Tcf binding sites, Upstate Biotechnology) or FOPFLASH (2 µg, containing inactive Tcf binding sites, Upstate Biotechnology) and pCH110 (0.2 µg, containing β-galactosidase, GE Healthcare) as control of transfection efficiency using Lipofectamine 2000 as described above. Cell lysates were prepared 3 days after transfection and luciferase activity was measured with Dual-Light® System (Applied Biosystems). To deplete CS chains, cells were incubated in the presence of 100 mU/ml ChABC during cell culture. We confirmed the reduction of CS structure by FACS analysis (Figure S8). Luminescence was measured with a Lumat LB9501 luminometer (Berthold). Luciferase activity was normalized for transfection efficiency by β-galactosidase activity. Relative luciferase activity is defined as the ratio of luciferase activity of TOPFLASH to that of FOPFLASH.

### Immunostaining

Six days after replating of EBs on PLL/laminin-coated glass chamber slides (Iwaki) to induce neuronal differentiation, cells were fixed with 4% paraformaldehyde and permeabilized with 0.1% saponin. After washing and subsequent blocking, cells were stained with an anti-βIII-Tubulin antibody. After washing, cells were stained with an FITC-conjugated secondary antibody and counterstained with PI. Immunofluorescence images were obtained using an LSM5Pascal confocal laser scanning microscope (Carl Zeiss).

### SPR Analysis

Sugar Chips immobilized with heparin (Nacalai Tesque) or CS-E (Seikagaku Corp.) were purchased from SUDx-Biotec (Kagoshima, Japan) prepared as previously described [17] and were set on a prism with refraction oil (n_D_ = 1.518, Cargill Laboratories Inc.) in an SPR apparatus (SPR670M, Moritex, Yokohama, Japan). The SPR measurements were performed at room temperature in accordance with the manufacturer's instructions and using Tris buffered saline (20 mM Tris-HCl, 150 mM NaCl, pH 7.4) containing 0.05% Tween-20 and 0.1% BSA as the running buffer at a flow rate of 15 µl/min. The kinetic binding parameters were calculated using the software of the manufacturer.

### RT-PCR and Real Time PCR

Total RNA was isolated from cells by TRIZOL Reagent (Invitrogen) and subsequently reverse transcribed using an oligo-dT primer (Invitrogen) and a SuperscriptII first strand synthesis kit (Invitrogen). Real time PCR was performed using an ABI PRISM® 7700 sequence detection system (Applied Biosystems). The relative amounts of each mRNA were normalized by *β-actin* mRNA in the same cDNA. Primer sets for RT-PCR and primer sets and probes for real time PCR are listed in Tables S2, S3 and S4, respectively.

## Supporting Information

Figure S1 PAPS transport activity. The results are shown after normalization against the values obtained with control cells (value = 1). The values shown are the means±SD of three independent experiments and significant values are indicated; *P<0.05, in comparison to the control.
(0.14 MB TIF)

Figure S2 (A) Photomicrographs of cells 4 days after transfection. Representative photographs of control and *PAPST*-KD cells from two independent experiments are shown. Scale bar, 50 µm. (B) Real time PCR analysis of germ layer markers (*Gata6*, primitive endoderm; *LamininB1*, parietal endoderm; *Bmp2*, visceral endoderm; *Cdx2*, trophoblast; *Fgf-5*, primitive ectoderm; *Isl1*, neuroectoderm; *Brachyury*, mesoderm) in the cells 4 days after transfection. The results are shown after normalization against the values obtained with control cells (value = 1). The values shown are the means±SD from two independent experiments. (*open bars*, control cells; *solid bars*, from left, *PAPST1*-KD, *PAPST2*-KD, *PAPST1*+*2*-KD, and *NDST1*+*2*-KD cells, respectively).
(2.40 MB TIF)

Figure S3 (A) Self-renewal assay. The ratio of alkaline phosphatase positive colonies is shown after normalization against the ratio obtained with non-treated cells (value = 1). The values shown are the means±SD from three independent. (B) Proliferation assay. The ratio of proliferation 48 h after culture is shown after normalization against the values obtained with non-treated cells (value = 1). The values shown are the means±SD from three independent experiments. (C) Luciferase reporter assay. Relative luciferase activities (TOPFLASH/FOPFLASH) are shown as means±SD from three independent experiments after normalization against the values obtained with non-treated cells (value = 1). In (A) – (C), cells were incubated in the presence of 100 mU/ml ChABC during cell culture. We confirmed the reduction of CS structure by FACS analysis (Figure S8).
(0.64 MB TIF)

Figure S4 (A) RT-PCR analysis of the expression of several *FGFs* and *FGFRs* in mESCs and MEFs. (B) Proliferation assay. The ratio of proliferation 48 h after culture is shown after normalization against the values obtained with DMSO-treated cells (value = 1). The values shown are the means±SD from three independent experiments and significant values are indicated; *P<0.01, in comparison to DMSO-treated cells.
(1.98 MB TIF)

Figure S5 Western blot analysis of cells 3 days after transfection. Representative immunoblots are shown. The histograms show mean densitometric readings±SD of β-catenin/Lamin B1 after normalization against the values obtained with control cells (value = 1). Values were obtained from duplicate measurements of two independent experiments and significant values are indicated; *P<0.01, in comparison to the control.
(0.52 MB TIF)

Figure S6 Western blot analysis 6 days after replating of EBs. Representative immunoblots are shown. The histograms show mean densitometric readings±SD of βIII-Tubulin/β-actin after normalization against the values obtained with control cells not treated with RA (value = 1). Values were obtained from duplicate measurements of two independent experiments and significant values are indicated; *P<0.03, in comparison to the control.
(0.84 MB TIF)

Figure S7 (A) FACS analysis of RA-treated and non-treated EBs using an anti-CS antibody or anti-HS (HepSS-1) antibody. Black line represents IgM isotype control. Three independent experiments were performed and representative results are shown. (B) FACS analysis of RA-treated EBs using an anti-HNK-1 antibody. Black line represents IgM isotype control. Three independent experiments were performed and representative results are shown.
(0.61 MB TIF)

Figure S8 (A) FACS analysis of heparitinase-treated or ChABC-treated mESCs using an anti-HS (10E4) antibody or anti-CS antibody. Black line represents IgM isotype control. Three independent experiments were performed and representative results are shown.
(0.35 MB TIF)

Table S1 The single-stranded DNA oligonucleotide sequence
(0.05 MB DOC)

Table S2 List of gene specific primers for RT-PCR
(0.05 MB DOC)

Table S3 List of gene specific primers for real time PCR
(0.06 MB DOC)

Table S4 List of gene specific probes for real time PCR
(0.05 MB DOC)
