# Supplementary figures and images for: The 3′-Phosphoadenosine 5′-Phosphosulfate Transporters, PAPST1 and 2, Contribute to the Maintenance and Differentiation of Mouse Embryonic Stem Cells
Source: PLoS One. 2009 Dec 11;4(12):e8262. doi: 10.1371/journal.pone.0008262 (PMC2788424; doi:10.1371/journal.pone.0008262)

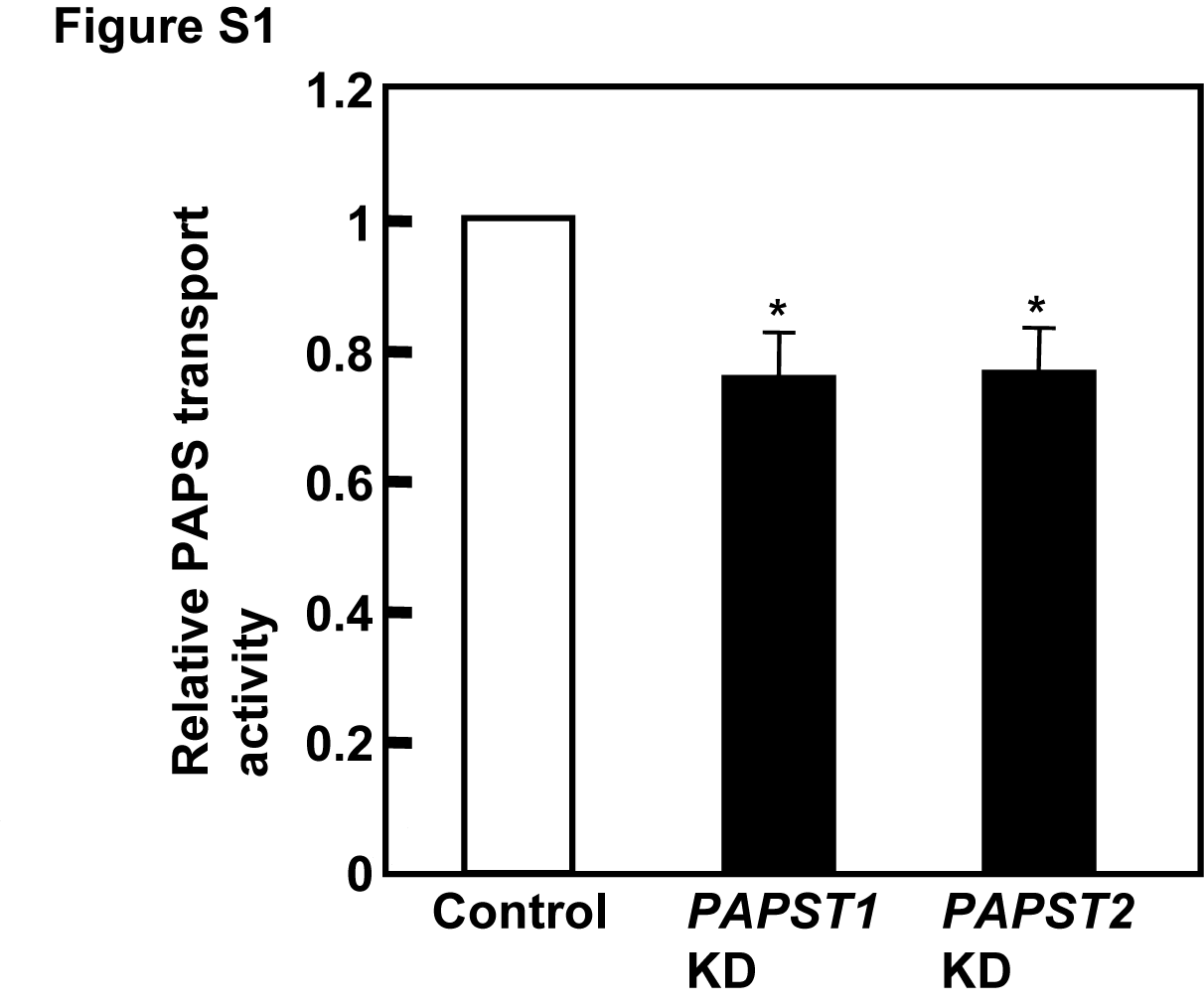

Supplement: Figure S1 — PAPS transport activity. The results are shown after normalization against the values obtained with control cells (value = 1). The values shown are the means±SD of three independent experiments and significant values are indicated; *P<0.05, in comparison to the control. (0.14 MB TIF) [file pone.0008262.s001.tif]

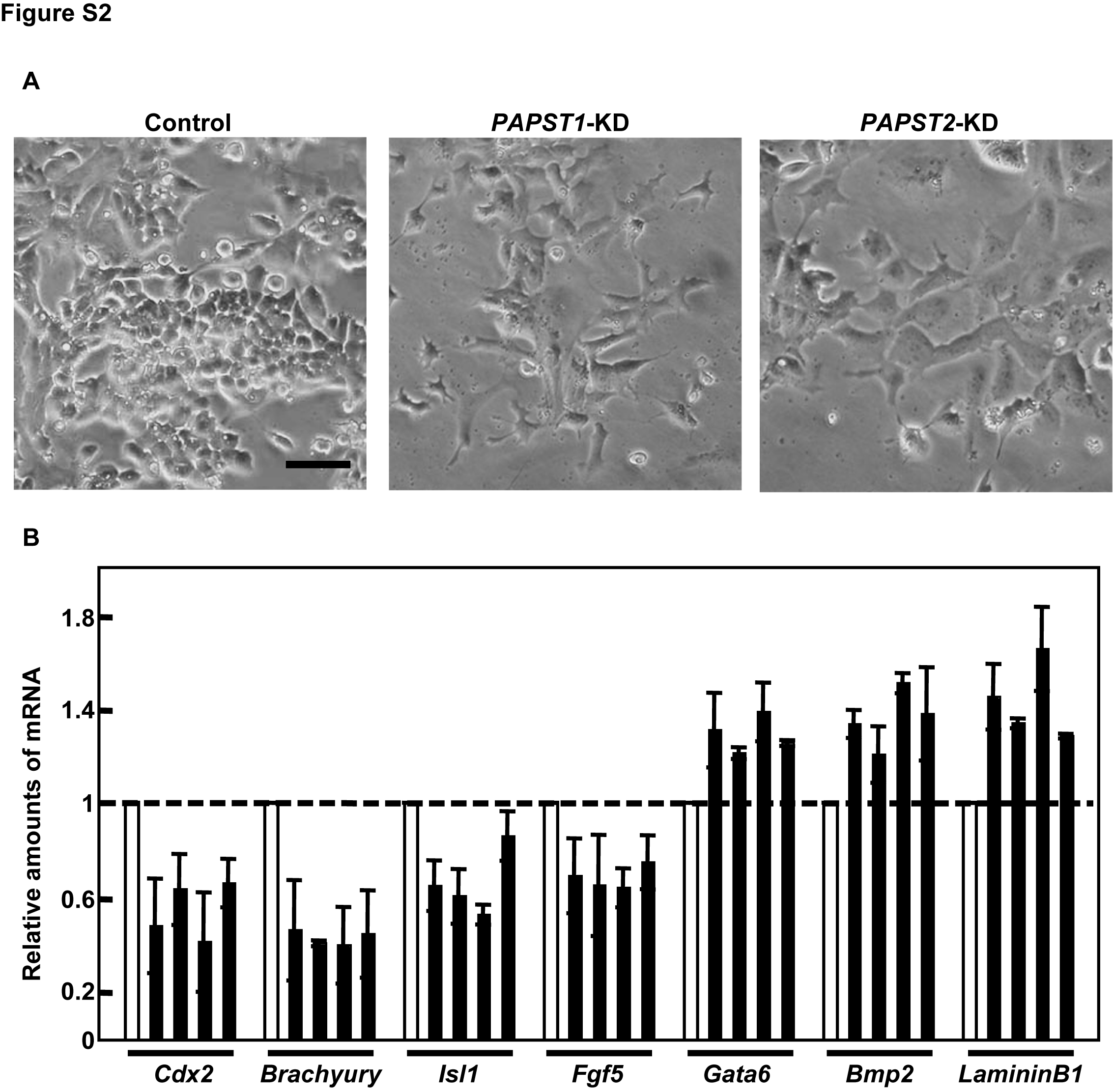

Supplement: Figure S2 — (A) Photomicrographs of cells 4 days after transfection. Representative photographs of control and PAPST-KD cells from two independent experiments are shown. Scale bar, 50 µm. (B) Real time PCR analysis of germ layer markers (Gata6, primitive endoderm; LamininB1, parietal endoderm; Bmp2, visceral endoderm; Cdx2, trophoblast; Fgf-5, primitive ectoderm; Isl1, neuroectoderm; Brachyury, mesoderm) in the cells 4 days after transfection. The results are shown after normalization against the values obtained with control cells (value = 1). The values shown are the means±SD from two independent experiments. (open bars, control cells; solid bars, from left, PAPST1-KD, PAPST2-KD, PAPST1+2-KD, and NDST1+2-KD cells, respectively). (2.40 MB TIF) [file pone.0008262.s002.tif]

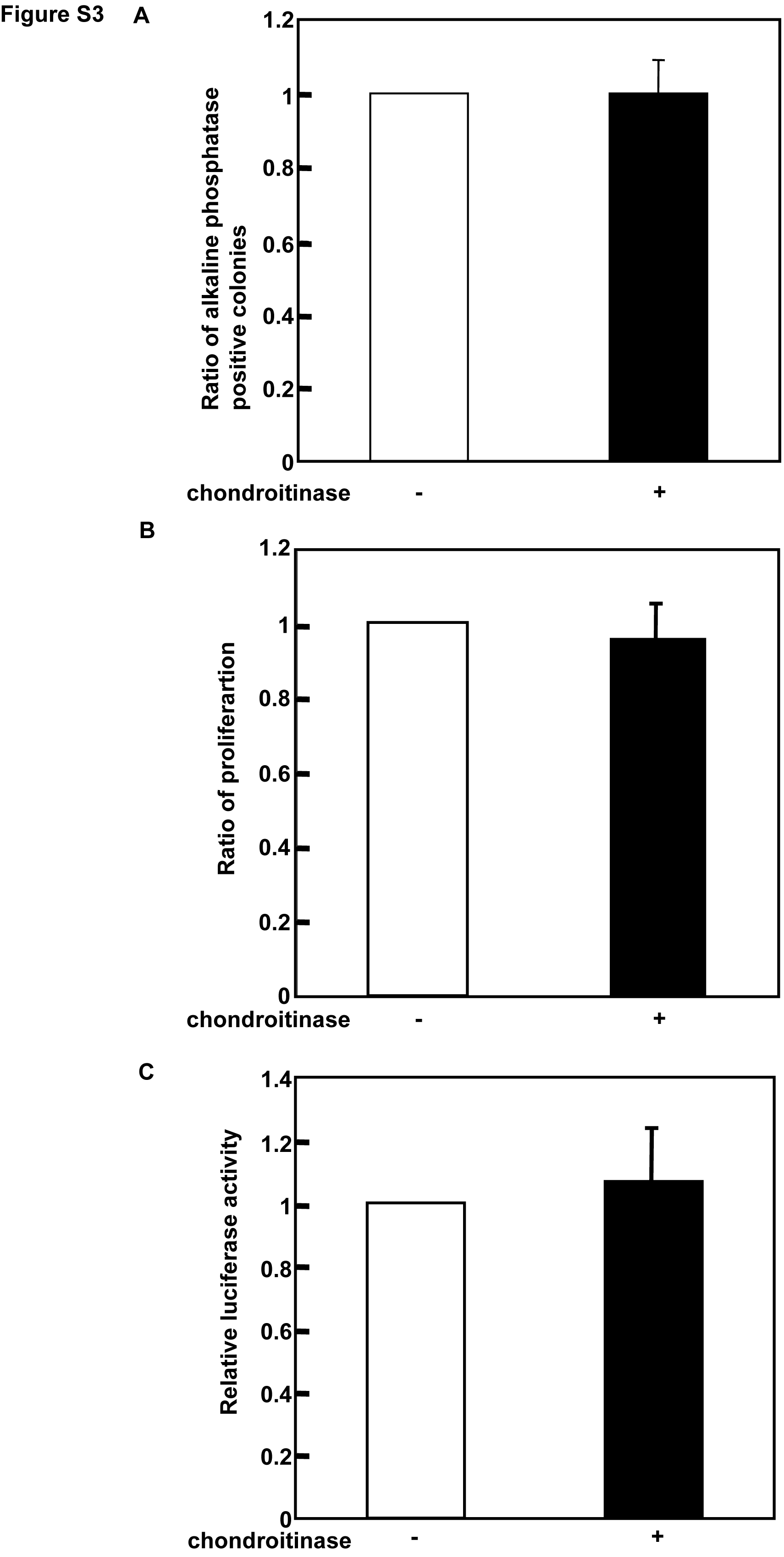

Supplement: Figure S3 — (A) Self-renewal assay. The ratio of alkaline phosphatase positive colonies is shown after normalization against the ratio obtained with non-treated cells (value = 1). The values shown are the means±SD from three independent. (B) Proliferation assay. The ratio of proliferation 48 h after culture is shown after normalization against the values obtained with non-treated cells (value = 1). The values shown are the means±SD from three independent experiments. (C) Luciferase reporter assay. Relative luciferase activities (TOPFLASH/FOPFLASH) are shown as means±SD from three independent experiments after normalization against the values obtained with non-treated cells (value = 1). In (A) – (C), cells were incubated in the presence of 100 mU/ml ChABC during cell culture. We confirmed the reduction of CS structure by FACS analysis (Figure S8). (0.64 MB TIF) [file pone.0008262.s003.tif]

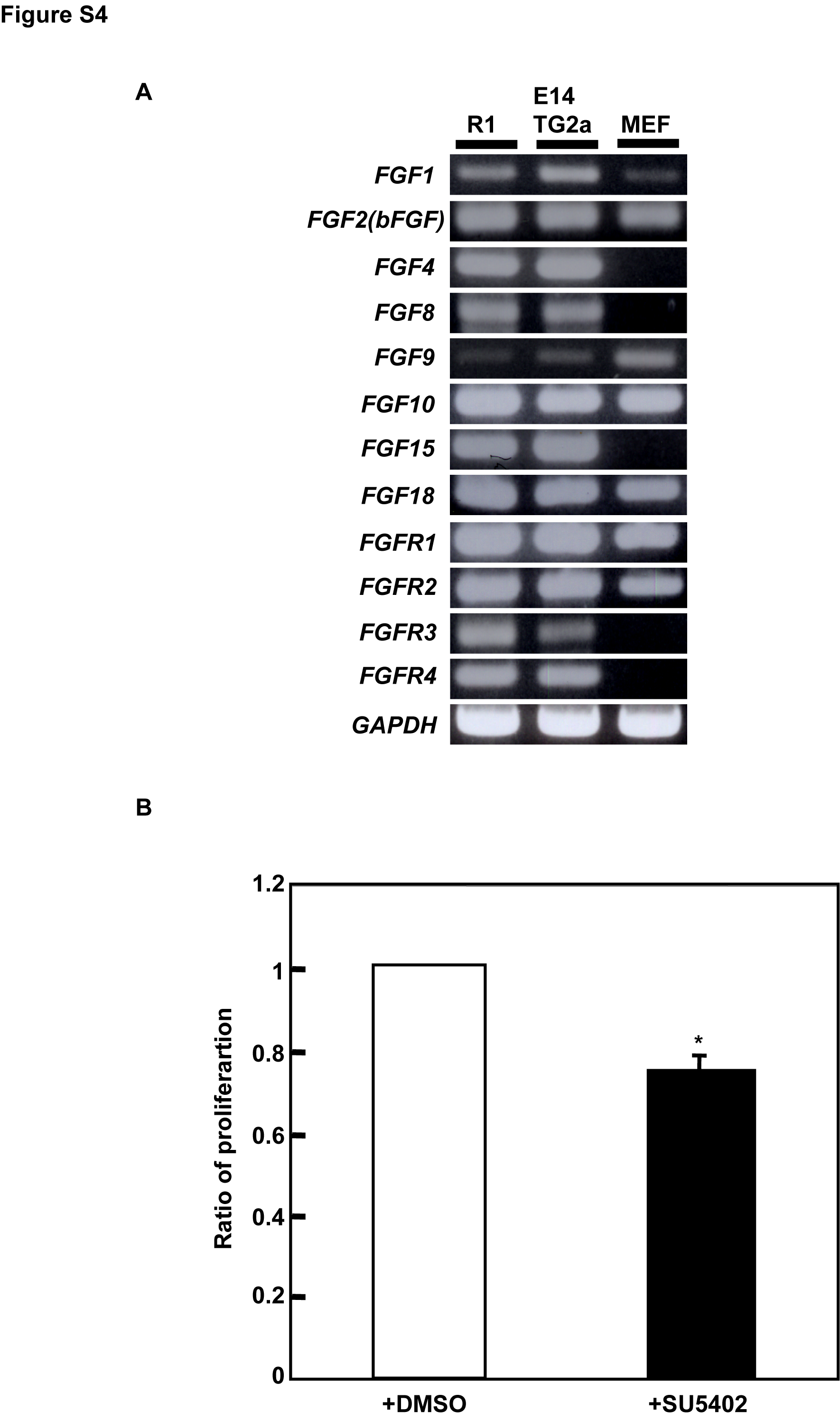

Supplement: Figure S4 — (A) RT-PCR analysis of the expression of several FGFs and FGFRs in mESCs and MEFs. (B) Proliferation assay. The ratio of proliferation 48 h after culture is shown after normalization against the values obtained with DMSO-treated cells (value = 1). The values shown are the means±SD from three independent experiments and significant values are indicated; *P<0.01, in comparison to DMSO-treated cells. (1.98 MB TIF) [file pone.0008262.s004.tif]

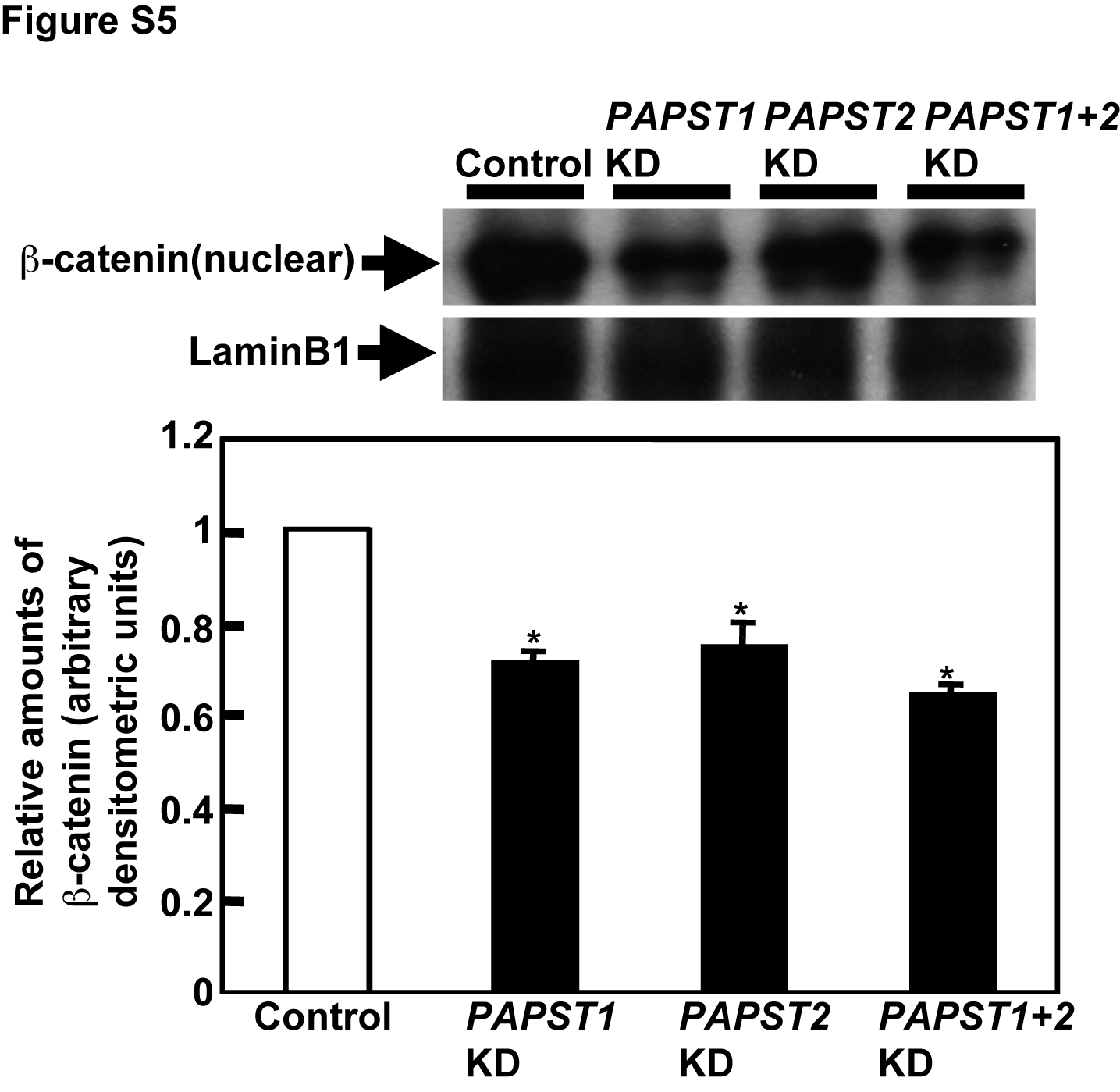

Supplement: Figure S5 — Western blot analysis of cells 3 days after transfection. Representative immunoblots are shown. The histograms show mean densitometric readings±SD of β-catenin/Lamin B1 after normalization against the values obtained with control cells (value = 1). Values were obtained from duplicate measurements of two independent experiments and significant values are indicated; *P<0.01, in comparison to the control. (0.52 MB TIF) [file pone.0008262.s005.tif]

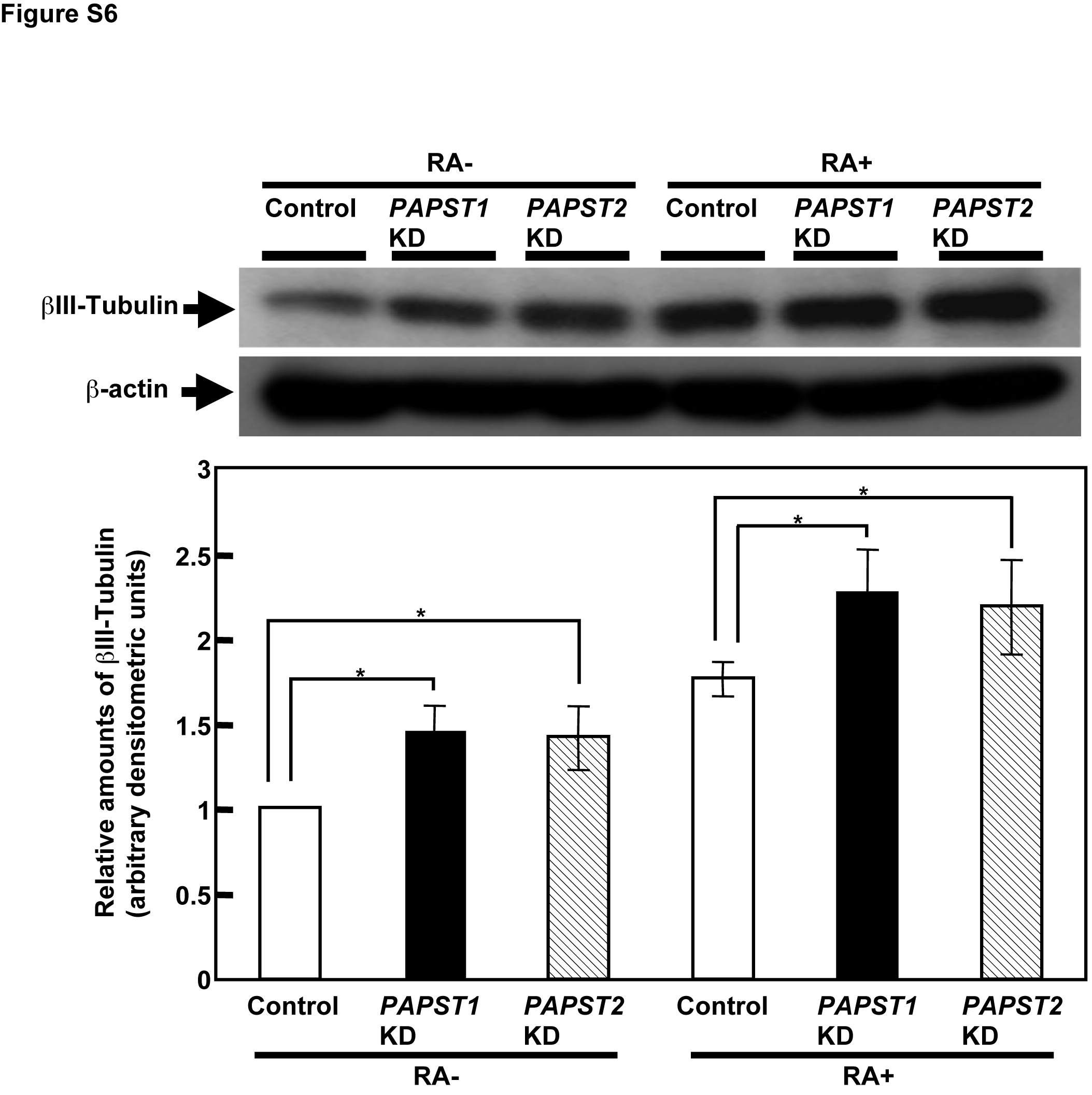

Supplement: Figure S6 — Western blot analysis 6 days after replating of EBs. Representative immunoblots are shown. The histograms show mean densitometric readings±SD of βIII-Tubulin/β-actin after normalization against the values obtained with control cells not treated with RA (value = 1). Values were obtained from duplicate measurements of two independent experiments and significant values are indicated; *P<0.03, in comparison to the control. (0.84 MB TIF) [file pone.0008262.s006.tif]

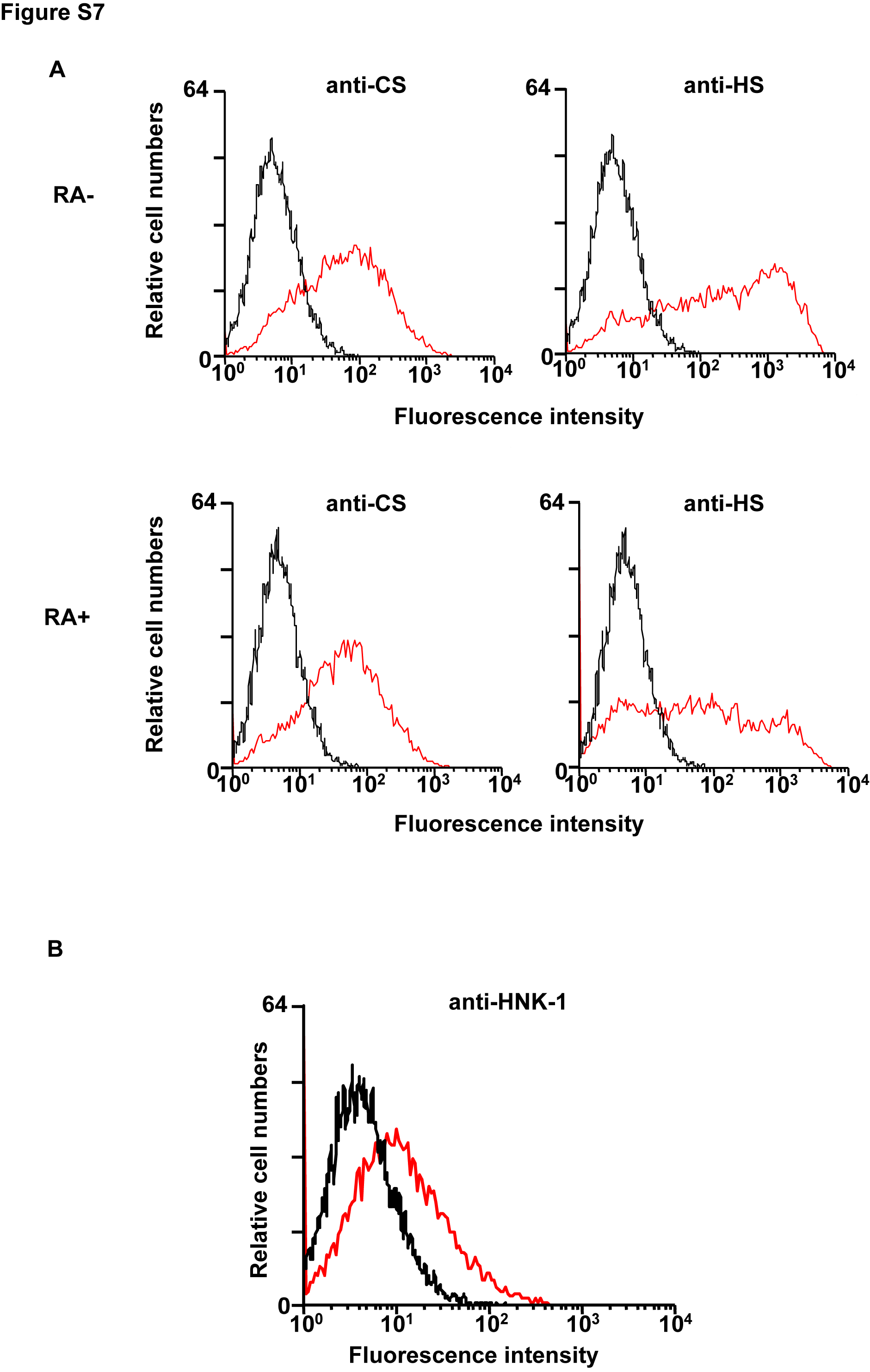

Supplement: Figure S7 — (A) FACS analysis of RA-treated and non-treated EBs using an anti-CS antibody or anti-HS (HepSS-1) antibody. Black line represents IgM isotype control. Three independent experiments were performed and representative results are shown. (B) FACS analysis of RA-treated EBs using an anti-HNK-1 antibody. Black line represents IgM isotype control. Three independent experiments were performed and representative results are shown. (0.61 MB TIF) [file pone.0008262.s007.tif]

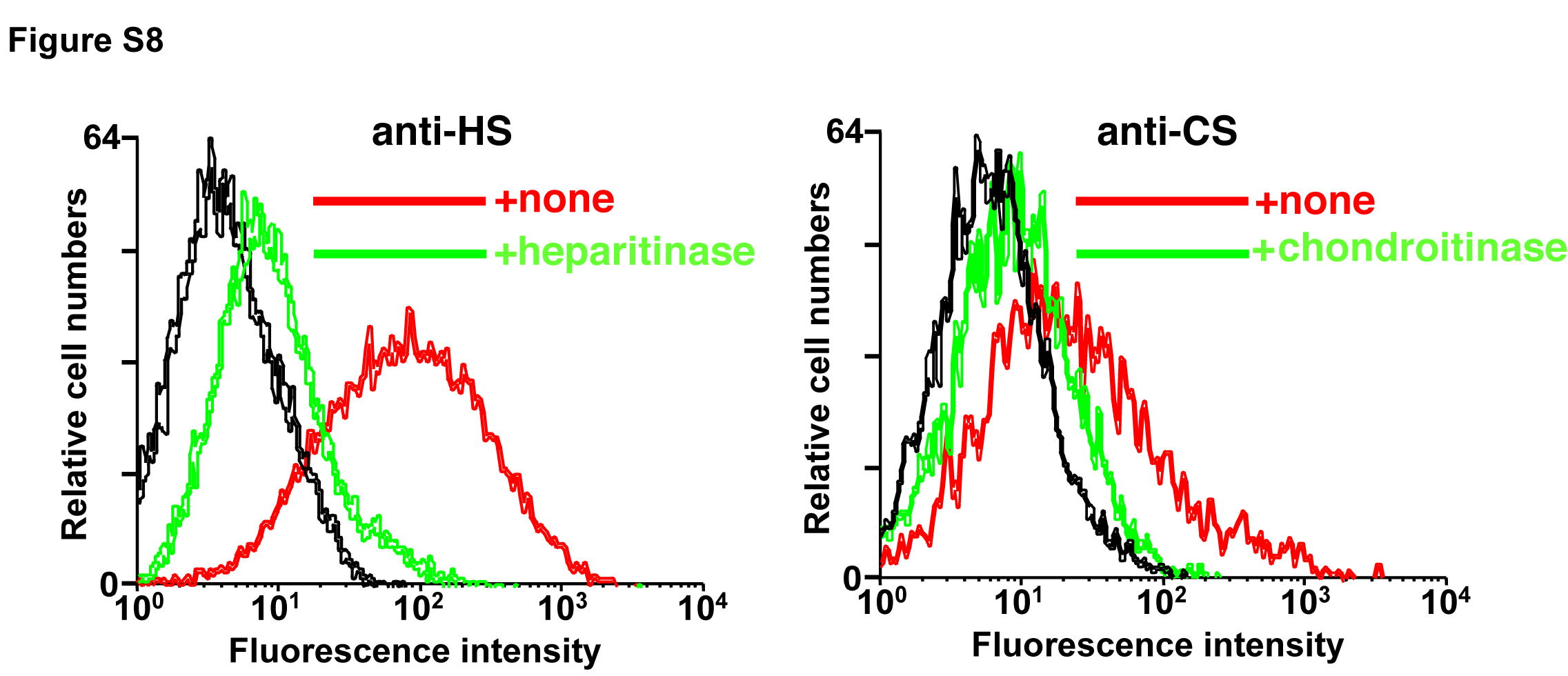

Supplement: Figure S8 — (A) FACS analysis of heparitinase-treated or ChABC-treated mESCs using an anti-HS (10E4) antibody or anti-CS antibody. Black line represents IgM isotype control. Three independent experiments were performed and representative results are shown. (0.35 MB TIF) [file pone.0008262.s008.tif]
