# Supplementary material for: The 3′-Phosphoadenosine 5′-Phosphosulfate Transporters, PAPST1 and 2, Contribute to the Maintenance and Differentiation of Mouse Embryonic Stem Cells
Source: PLoS One. 2009 Dec 11;4(12):e8262. doi: 10.1371/journal.pone.0008262 (PMC2788424; doi:10.1371/journal.pone.0008262)
Supplement: Table S1 — The single-stranded DNA oligonucleotide sequence (0.05 MB DOC) [file pone.0008262.s009.doc]

| **Gene** |  |
| --- | --- |
| *EGFP* | 5’-GATCCCGCCACAACGTCTATATCATGG**GGAAAAT**CCATGATATAGACGTTGT  GGCTTTTTTGGAAA-3’ |
| *PAPST1-1* | 5’-GATCCCCGGGCTATGCCAGCTTTATGG**GCTTCCTGTCAC**CCATAAAGCTGG  CATAGCCCGTTTTTTGGAAA-3’ |
| *PAPST1-2* | 5’-GATCCCCCTGGTGCAGTACTTAAGACG**GCTTCCTGTCAC**CGTCTTAAGTAC  TGCACCAGGTTTTTTGGAAA-3’ |
| *PAPST2-1* | 5’-GATCCCCCATCGGTGTACAACATGATA**GCTTCCTGTCAC**TATCATGTTGTAC  ACCGATGGTTTTTTGGAAA–3’ |
| *PAPST2-2* | 5’-GATCCCCACAATTGCACCAAACTTTAA**GCTTCCTGTCAC**TTAAAGTTTGGT  GCAATTGTGTTTTTTGGAAA–3’ |
| *NDST1-1* | 5’-gatcccCCCTAGTGTTGCCCTAAGAGG**gcttcctgtcac**CCTCTTAGGGCA  ACACTAGGGttttttggaaa-3’ |
| *NDST1-2* | 5’-gatcccCGGTGCGCATAGGGAATGAGG**gcttcctgtcac**CCTCATTCCCTAT  GCGCACCGttttttggaaa-3’ |
| *NDST2-1* | 5’-gatcccCTTAATCCGATCAGTGTCTTT**gcttcctgtcac**AAAGACACTGATC  GGATTAAGttttttggaaa-3’ |
| *NDST2-2* | 5’-gatcccCGCTACATCTTAGTAGATATC**gcttcctgtcac**GATATCTACTAAGA  TGTAGCGttttttggaaa-3’ |

The *underlined* 21 nucleotides are sense and antisense siRNA target sequences, respectively. The *boldface* nucleotides are loop sequences.
