# Supplementary material for: The 3′-Phosphoadenosine 5′-Phosphosulfate Transporters, PAPST1 and 2, Contribute to the Maintenance and Differentiation of Mouse Embryonic Stem Cells
Source: PLoS One. 2009 Dec 11;4(12):e8262. doi: 10.1371/journal.pone.0008262 (PMC2788424; doi:10.1371/journal.pone.0008262)
Supplement: Table S2 — List of gene specific primers for RT-PCR (0.05 MB DOC) [file pone.0008262.s010.doc]

| **Gene** | **Forward primer** | **Reverse primer** |
| --- | --- | --- |
| *FGF1* | AAAGTGGAGTGAAGAGAGCC | CTTTCTGGCCATAGTGAGTC |
| *FGF2* | CCCACACGTCAAACTACAACT | GCAGACATTGGAAGAAACAG |
| *FGF4* | ACTACCTGCTGGGCCTCAAA | TTCATGGTAGGCGACACTCG |
| *FGF8* | TGTTGCACTTGCTGGTTCTC | CCAAAAGTATCGGTCTCCAC |
| *FGF9* | ACCCTTTTTCCCTCTCTGTC | GTTCCCTGGATAGTACCGTT |
| *FGF10* | CACCAACTGCTCTTCTTCCT | GCCATTGTGCTGCCAGTTAA |
| *FGF15* | CCCTATGTCTCCAACTGCTT | AAGAAGGAGCGGTGAAACAC |
| *FGF18* | GAGATGATGTGAGTCGGAAG | TACATCTTGCTGGTTCTCGC |
| *FGFR1* | AATGTCTCAGATGCACTCCC | AGCTGGTAGGTGTGGTTGAT |
| *FGFR2* | CGTCAGACAAAGGCAACTAC | GGCGATTAAGAAGACCCCTA |
| *FGFR3* | AAGGATGGTACAGGTCTGGT | ACACAGGTATAGTTGCCACG |
| *FGFR4* | CCATGACCGTCGTACACAAT | CGAATGCTACCCAGAGAGTT |
| *NDST1* | GCAAGGAGGGCACACGC | GCGGAGTTCATTCTGTGTATCAAA |
| *NDST2* | CGCTGGCTGACTTACTATCCCT | CCGGGTTGACACGAAGCT |
| *GAPDH* | AGGTCGGTGTGAACGGATTT | AGAGTGGGAGTTGCTGTTGA |
