# Supplementary material for: The 3′-Phosphoadenosine 5′-Phosphosulfate Transporters, PAPST1 and 2, Contribute to the Maintenance and Differentiation of Mouse Embryonic Stem Cells
Source: PLoS One. 2009 Dec 11;4(12):e8262. doi: 10.1371/journal.pone.0008262 (PMC2788424; doi:10.1371/journal.pone.0008262)
Supplement: Table S3 — List of gene specific primers for real time PCR (0.06 MB DOC) [file pone.0008262.s011.doc]

| **Gene** | **Forward primer** | **Reverse primer** |
| --- | --- | --- |
| *PAPST1* | GGACAGAGACAGCGGAATC | ACCCGAGGCACAGAAGACC |
| *PAPST2* | CAGGAGAAGGCCATGAAACTG | CACAAACCCGATTGAGTAGGAAT |
| *Oct3/4* | CTCACCCTGGGCGTTCTCT | AGGCCTCGAAGCGACAGA |
| *Nanog* | CAGAAAAACCAGTGGTTGAAGACTAG | GCAATGGATGCTGGGATACTC |
| *Gata6* | CCCCTCATCAAGCCACAGAA | GTGACAGTTGGCACAGGACAGT |
| *LamininB1* | GCAGAAAGCCATGGATTTTGA | CAGAGACCATCTTGGAGAGCTGTT |
| *Bmp2* | AAAGCGTCAAGCCAAACACAA | AGTCCACATACAAAGGGTGTCTCTT |
| *Cdx2* | GAGCTGGCTGCCACACTTG | GCTTCTTCTTGATTTTCCTCTCCTT |
| *Fgf5* | GCAGCCCACGGGTCAA | CGGTTGCTCGGACTGCTT |
| *Isl1* | ATCGAGTGTTTCCGCTGTGTAG | TCCCGCAGGGCGAATT |
| *Brachyury* | TGCTGCAGTCCCATGATAACTG | ATGACTCACAGGCAGCATGCT |
| *Mash1* | CCTCCCCAAACCCCACAT | ACAAAGATTGGAAGCATTCTATGAAG |
| *Pax6* | AACCTGGCTAGCGAAAAGCA | CCCGTTCAACATCCTTAGTTTATCA |
| *Goosecoid* | AGCCAAGTGGAGACGACAGAA | TTGAGGACGTCTTGTTCCACTTC |
| *Nestin* | TGCAGACACCTGGAAGAAGTTC | CCCAAGGAAATGCAGCTTCA |
| *Musashi-1* | GGTGGCCTTTCCTCGAAGAG | GCCCCCCCACGAAGATC |
| *Math1* | ATGCACGGGCTGAACCA | TCGTTGTTGAAGGACGGGATA |
| *NeuroD1* | CCAGCCCACTACCAATTTGG | GGGTTCTGCTCAGGCAAGAA |
| *NeuroD2* | GCAGAAGCTGTCCAAGATCGA | AGCGCAAGATCTCCGAGAGA |
| *β-actin* | GCTCTGGCTCCTAGCACCAT | GCCACCGATCCACACAGAGT |
