# Supplementary material for: The 3′-Phosphoadenosine 5′-Phosphosulfate Transporters, PAPST1 and 2, Contribute to the Maintenance and Differentiation of Mouse Embryonic Stem Cells
Source: PLoS One. 2009 Dec 11;4(12):e8262. doi: 10.1371/journal.pone.0008262 (PMC2788424; doi:10.1371/journal.pone.0008262)
Supplement: Table S4 — List of gene specific probes for real time PCR (0.05 MB DOC) [file pone.0008262.s012.doc]

| **Gene** | **probe*^a^*** |
| --- | --- |
| *PAPST1* | CCCCGTCTTGGCAGGTCCTGA |
| *PAPST2* | ACAATGCTTCCAACTCGGAGATGGTTTTG |
| *Oct3/4* | TGGAAAGGTGTTCAGCCAGACCACC |
| *Nanog* | GTGCTGAGCCCTTCTGAATCAGACCATT |
| *Gata6* | CGTGCCTTCATCACGGCGGC |
| *LamininB1* | GACTTCAGCCAGGGCACTCAGGACA |
| *Bmp2* | ACAGCGGAAGCGCCTCAAGTCCA |
| *Cdx2* | GAAACCAAATTTTAACCTGCCTCTCGGAGAG |
| *Fgf5* | CCCCAACACGTCTCCACCCACTTC |
| *Isl1* | TGCAGCCGACAGCTCATCCCG |
| *Brachyury* | TCTAGCCTCGGAGTGCCTGGCCA |
| *Mash1* | CCCTCCCACGGTCTTTGCTTCTGTTT |
| *Pax6* | CATGCCGTCTGCGCCCATCTG |
| *Goosecoid* | CGGCGTTTTCTGACTCCTCCGAGG |
| *Nestin* | AGGCTTCTCTTGGCTTTCCTGACCCC |
| *Musashi-1* | TCTTCGTCCGAGTGACCATCTTAGGCTGT |
| *Math1* | CCTTCGACCAGCTGCGCAACG |
| *NeuroD1* | TGCCTGCAGCTCAACCCTCGG |
| *NeuroD2* | ACCCTGCGCCTGGCCAAGAACTAC |
| *β-actin* | ATCAAGATCATTGCTCCTCCTGAGCGC |

*^a^* The probe was labeled at the 5’-end with the reporter dye, 3FAM, and at the 3’-end with the quencher dye, TAMRA (Applied Biosystems).
